# Subicular Astrocytes Govern Seizure‐Impaired Fear Memory

**DOI:** 10.1002/advs.202510818

**Published:** 2025-11-20

**Authors:** Yuying Shao, Jing Xi, Yuhao Sun, Yulan Li, Zhisheng Li, Wangjialu Lu, Jialu Chen, Lin Yang, Fan Fei, Heming Cheng, Li Cheng, Cenglin Xu, Zhuo Huang, Vladimir Parpura, Yi Wang, Zhong Chen

**Affiliations:** ^1^ Key Laboratory of Medical Neurobiology of the Ministry of Health of China Institute of Pharmacology & Toxicology College of Pharmaceutical Sciences School of Medicine Zhejiang University Hangzhou 310058 China; ^2^ Zhejiang Collaborative Innovation Center for the Brain Diseases with Integrative Medicine Zhejiang Key Laboratory of Neuropsychopharmacology School of Pharmaceutical Sciences Zhejiang Chinese Medical University Hangzhou 310053 China; ^3^ State Key Laboratory of Natural and Biomimetic Drugs Department of Molecular and Cellular Pharmacology School of Pharmaceutical Sciences Peking University Health Science Center Beijing 100191 China; ^4^ International Translational Neuroscience Research Institute School of Pharmaceutical Sciences Zhejiang Chinese Medical University Hangzhou 310053 China

**Keywords:** astrocytes, epilepsy, fear memory, seizure, subiculum

## Abstract

The mechanisms underlying seizure‐associated cognitive impairment remain incompletely characterized. Emerging evidence positions the subiculum, a hippocampal output hub critically involved in both epileptic seizure and cognitive performance, as a putative nexus for this comorbidity. Here, it is demonstrated that astrocytic activation in the subiculum mediates seizure‐induced fear memory deficits. Subicular astrocytes dynamically respond to conditioned fear memory learning, acting as a “scavenger” for the inhibition of context memory. Seizure activity hyperactivates these leaning‐associated astrocytes and amplifies their engagement during fear processing. Suppression of subicular astrocyte Ca^2+^ signaling fully rescues seizure‐induced fear memory deficits, while Gq pathway activation in the subicular astrocytes replicates cognitive impairment. Mechanistically, this seizure‐induced astrocyte dysregulation specifically involves Ca^2+^‐dependent gliotransmitter adenosine‐mediated inhibition through A_1_ receptors, reducing local neuronal excitability during fear processing. Collectively, these findings identify subicular astrocytes as critical modulators of seizure‐associated cognitive dysfunction, operating through a Ca^2+^‐dependent adenosine‐linked pathway that disrupts neural circuit homeostasis. This work highlights the potential for astrocyte‐targeted interventions as a therapeutic strategy for seizure‐related memory disorders.

## Graphical abstract

Astrocytes in dorsal subiculum act as a critical modulator of seizure‐associated cognitive dysfunction, operating through a Ca^2+^‐dependent adenosine‐linked astrocyte‐neuron signaling pathway that disrupts neuronal circuit homeostasis. This research highlights the potential of astrocyte‐targeted interventions as a therapeutic strategy, moving beyond the conventional neuron‐centric view for treating seizure‐related fear memory disorders.

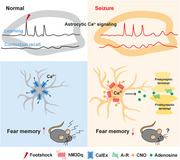

## 1 Introduction

Epilepsy is one of the most common and disabling brain disorders, affecting ≈1% of the population of all ages. This disorder is frequently accompanied by cognitive impairments.^[^ ^1^ ^]^ In chronic epilepsy, cognitive deficits are observed in ≈70–80% of patients, which profoundly impair the quality of life and remain refractory to conventional treatments.^[^ ^2^ ^]^ While neuronal loss and synaptic dysfunction in the hippocampus have long been implicated in these deficits, accumulating evidence highlights the limitations of neuron‐centric models, as current antiseizure medications fail to halt cognitive decline even when seizure control is achieved.^[^ ^3^ ^]^ This therapeutic gap underscores the urgent need to explore non‐neuronal mechanisms, particularly those involving glial cells, which dynamically regulate both epileptic seizures and cognitive networks.^[^ ^4^ ^]^

Astrocytes are no longer considered to merely provide homeostatic support to neurons and to encapsulate synapses, as pioneering research has shown that they can actively sense and modify synaptic activity as an integral part of the “tripartite synapse”.^[^ ^5^, ^6^, ^7^ ^]^ Recently, studies show that hippocampal CA1 astrocytes, as “engram cells”, actively participate in memory formation and retrieval in coordination with neuronal ensembles.^[^ ^8^, ^9^ ^]^ In the epileptic condition, unlike neurons, which fire transiently (electrical activity) during seizures, astrocytes exhibit persistent hyperactivity (based on intracellular Ca^2+^ dynamics and the display of a so‐called reactive astrogliosis) for days post‐ictally, aligning temporally with the progression of cognitive decline.^[^ ^10^, ^11^ ^]^ Compared to other hippocampal subregions, the subiculum, which collects the information emanating from the hippocampus and sends projections to cortical and subcortical areas,^[^ ^12^ ^]^ is relatively preserved but dysfunctional in epilepsy, making it a unique functional unit critical for seizure initiation and propagation.^[^ ^13^, ^14^, ^15^, ^16^ ^]^ Anatomically positioned to integrate spatial and contextual information, the subiculum coordinates hippocampal‐cortical communication essential for memory consolidation and retrieval.^[^ ^17^ ^]^ The subiculum also plays a central role in diverse cognitive processes, through its heterogeneous cellular composition and parallel circuit architectures—direct CA1‐entorhinal cortex (EC) projections and indirect CA1‐subiculum‐EC pathways—which exhibit structural and functional dissociation to support complementary cognitive regulation,^[^ ^14^, ^18^ ^]^ Notably, subicular atrophy correlates with the severity of memory impairment in temporal lobe epilepsy patients,^[^ ^19^ ^]^ suggesting that the subiculum may emerge as a critical nexus bridging seizure propagation and memory. However, how subicular astrocytes possess region‐specific mechanisms in encoding cognitive function in pathological hyperexcitability remains unresolved.

In this study, we employed the hippocampal kindling‐induced seizure model and a classical 3 day fear‐conditioning paradigm, coupled with in vivo fiber photometry, viral‐mediated approach, and pharmacology in mice, to dissect astrocyte‐mediated mechanisms of seizure‐associated cognitive deficits. We revealed that subicular astrocytes act as critical modulators of seizure‐associated fear memory deficits, operating through an intracellular Ca^2+^‐dependent, adenosine‐linked extracellular humoral pathway that disrupts neural circuit homeostasis. The pharmacological blockade of A_1_Rs is sufficient to reverse seizure‐mediated memory deficits. Collectively, our findings not only reveal the critical role of subicular astrocytes in seizure‐related cognitive impairment but also provide a potential therapeutic approach targeting subicular astrocyte‐neuron adenosine signaling.

## 2 Results

### 2.1 Seizure Potentiates Subicular Astrocyte Responses in Fear Memory

**Figure 1:**
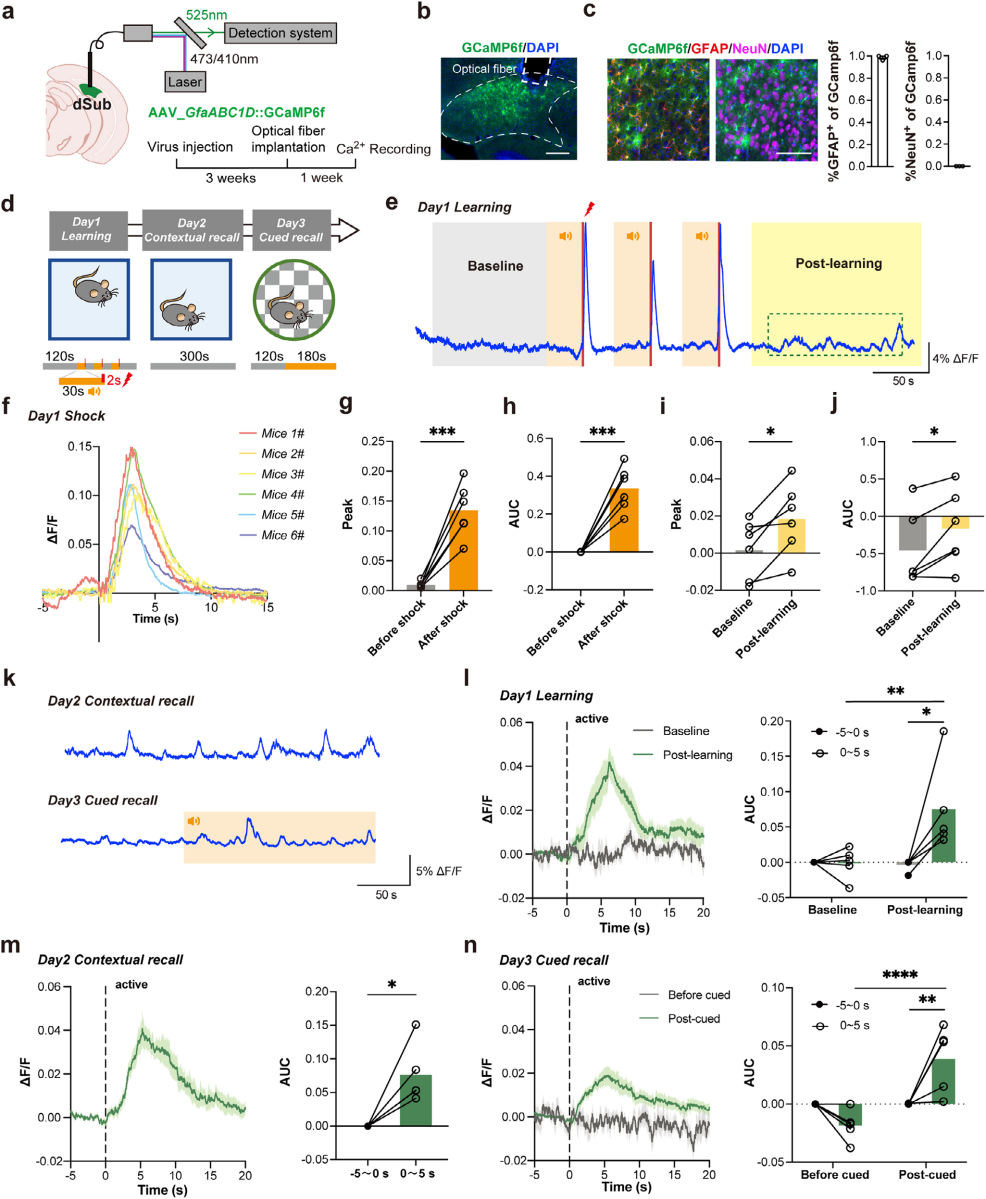
Subicular astrocytes are tonically activated after conditioned fear memory. a) Left, schematic of AAV5_*GfaABC1D*::GCaMP6f injection into the dorsal subiculum (dSub) and an optic fiber implantation above the dSub; Right, schematic of the light path (top) and experimental timeline (bottom). b) Representative image showing optic fiber placement (broken rectangle) and the Ca^2+^ indicator GCaMP6f cellular expression (green) with DAPI‐counterstained cell nuclei (blue) in the dSub (broken contour). Scale bar: 200 µm. c) Images: Immunofluorescence staining of GFAP (left) or NeuN (right) and fluorescence of GCaMP6f and DAPI. Scale bar: 100 µm. Graphs: Co‐localization analysis of the hippocampus shows astrocyte‐specific expression of GCaMP6f, as this signal co‐localizes almost to its entirety with that of glial fibrillary acidic protein (GFAP^+^ cells, i.e., astrocytes), while it cannot be detected in neurons (NeuN^+^). d) Schematic of the 3 day fear‐conditioning test. An orange bar with the speaker symbol indicates sound delivery, which in conditional stimulation is paired with the foot shock at the end. The novel context during Cued recall is indicated by the checkerboard e). Representative astrocytic cytosolic Ca^2+^ time series (ΔF/F percentage) for conditioned fear learning; 0.4 mA, 2 s foot shocks occurred at the 148, 208, and 268 s time points and terminated together with the paired 30 s sound stimulus, as indicated by vertical red lines. The orange rectangle represents the time range of sound stimulation, the gray rectangle represents the baseline range, and the yellow rectangle represents the post‐learning period. f) Traces of astrocytic Ca^2+^signals dynamics during shocks paired with sound. Results are based on 18 trials from 6 mice. g,h) Quantification of the peak and area under the curve (AUC) for Ca^2+^signals 5 s before and after shocks paired with sound (^***^P<0.001, paired *t*‐test, n=6). Results are based on 18 trials from 6 mice. i,j) Quantification of the peak and AUC of Ca^2+^ dynamics at baseline (20–120 s) and post‐learning (300–400 s) (^*^P<0.05, paired *t*‐test, n=6). k) Representative Ca^2+^time series (ΔF/F percentage) for contextual recall and cued recall, the orange rectangle represents the time range of sound stimulation. l–n) Left, quantification of Ca^2+^ signal activity at the initiation of exploratory behaviour (when mice move their body centers) during the 3 day fear‐conditioning test, with each event occurring at the vertical dashed line (time=0). Right, quantification of Ca^2+^ dynamics AUC 5 s before exploration and 5 s after exploration during the 3 day fear‐conditioning test. (^*^P<0.05, ^**^P<0.01, ^****^P<0.0001, two‐way ANOVA test with Šídák's multiple comparisons test; ^*^P<0.05, paired‐*t*‐test). Data are presented as mean ± SEM.

Calcium ion signaling often serves as an indicator of astrocytes' physiological and pathological activities.^[^ ^20^, ^21^ ^]^ In order to explore whether subicular astrocytes participate in conditioned fear memory, we employed fiber photometry to evaluate their real‐time Ca^2+^ dynamics in live mice. Using the adeno‐associated virus approach, we selectively expressed the genetically encoded cytosolic Ca^2+^ indicator GCaMP6f in subicular astrocytes under the control of *GfaABC1D* promoter, and then recorded the astrocytic cytosolic Ca^2+^ dynamics during the 3 day fear‐conditioning paradigm in vivo (**Figure** **1**a,b,d). After in vivo experiments, quantitative co‐localization analysis of the hippocampus demonstrated astrocyte‐specific viral tropism, with 98.7% infection efficiency in glial fibrillary acidic protein‐positive (GFAP^+^) cells, i.e., astrocytes, whereas no neuronal infection was detected in NeuN^+^ populations (Figure 1c). On the learning day (day 1), discrete shock‐evoked Ca^2+^ transients in vivo emerged with the precise temporal coupling to the electrical foot shock onset paired to the end of the sound epoch (Figure 1d,e). A Train of three such paired audio‐electrical stimuli (Figure 1d bottom) was used as the conditioned stimulus for the learning paradigm. Ca^2+^ transients in astrocytes exhibited stereotyped kinetics, characterized by rapid rise and decay, each within seconds (Figure 1f–h). Intriguingly, post‐learning, there was a significant increase in both the peak value and area under the curve (AUC) of spontaneous Ca^2+^ dynamics in the early consolidation period (Figure 1i,j). Further characterization revealed that these Ca^2+^ activity patterns were tightly associated with behavioral state transitions, specifically emerged during active locomotion (as indicated by center‐of‐mass displacement), and showed marked potentiation following unconditioned stimulus (foot shock only) presentations during associative learning (Figure 1k–n). No notable elevation was detected during a non‐associated environment exploration on day 3, i.e., during the cued recall (Figure 1n). These results demonstrated that subicular astrocytes exhibit extensive Ca^2+^ activity responses during fear acquisition, and that thereafter are tonically re‐activated after learning (early fear memory consolidation) and retrieval, temporally aligned with the initiation of active locomotion.

**Figure 2:**
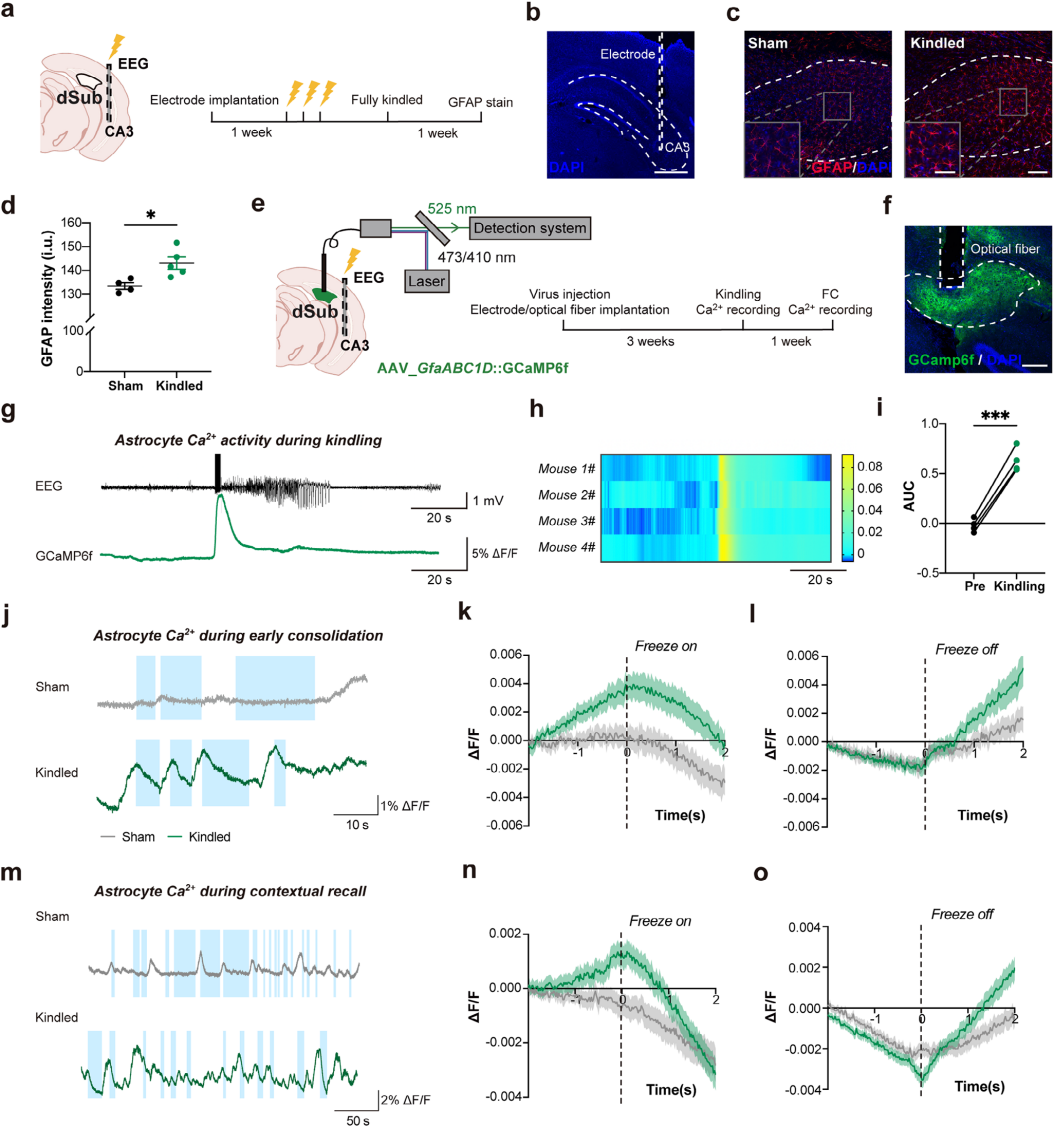
Seizures potentiate subicular astrocyte responses to conditioned fear memory. a) Left, schematic of electrode implantation into the right ventral hippocampal CA3 region; Right, schematic of the experiment; dSub, dorsal subiculum. b) Ventral hippocampal CA3 region (Dashed contour) slice with DAPI‐stained cell nuclei; verified electrode location (dashed vertical rectangle). Scale bar: 1 mm. c) Astrocytes in the CA3 region (dashed contour) were identified using GFAP (red) immunolabeling; cell nuclei were counterstained with DAPI. Close‐up of the areas indicated by grey rectangles is shown in the lower bottom corner insets. Scale bars: 100 µm in the full image and 50 µm in the inset. d) The quantification of the fluorescence intensity (GFAP), i.u., intensity units. e) Left, schematic of AAV5_*GfaABC1D*::GCaMP6f viral injection into dSub and optic fiber implantation above the dSub, and electrode, dual for stimulation and EEG recordings, implantation in the right ventral CA3 region; Right, schematic of the light path (top) and time‐frame of the experiment (bottom); FC, fear conditioning. f) Representative image showing optic fiber (vertical dashed rectangle) placement and astrocytic GCaMP6f expression (green) in the dSub (dashed contour). Scale bar: 200 µm. g) Representative EEG recording from the CA3 region (top) and Ca^2+^ signal traces (ΔF/F) of dSub astrocytes (bottom) during kindling. h) Left, heatmaps reporting ΔF/F of astrocytic GCaMP6f signals (n=4 mice, 3 trials per mouse). Right, vertical bar displays a pseudo color representation of ΔF/F ranging from 0 to 0.08. i) Quantification of the area under the curve (AUC) of astrocyte Ca^2+^ signals during kindling. (^***^P<0.001, paired *t*‐test). j) Representative Ca^2+^ signal traces (ΔF/F) from dSub astrocytes during early consolidation. The blue rectangles represent the periods of freezing behavior. k, l) The quantification of astrocytic Ca^2+^ signals (color coded as in j) at the initiation and termination of freezing behaviour during the early consolidation, with each freezing event occurring at the dashed line (time=0). For the sham group, 16 trials from 4 mice; for the kindled group, 20 trials from 4 mice. m) Representative Ca^2+^ ΔF/F traces of dSub astrocytes during the contextual recall. The blue rectangles as in j. n, o) The quantification of astrocyte Ca^2+^ signals as in (k, l) but during the contextual recall; dashed vertical line as in (k). For the sham group, 78 trials from 4 mice; for the kindled group, 68 trials from 4 mice. Data are presented as mean ± SEM.

To investigate the involvement of subicular astrocytes in fear memory following seizures, we implemented the mouse hippocampal kindling model. This is an ideal model in seizure‐related cognition impairment as it damages the mouse's contextual memory.^[^ ^22^ ^]^ Here, individual electrodes were implanted into the right ventral CA3 and dually used for electroencephalographic (EEG) recordings and electrical stimulation of mice until fully kindled (**Figure** **2**a,b). In the sham group of mice, implanted electrodes were not stimulated. One week later, mice were sacrificed, and we conducted GFAP immunofluorescence staining (Figure 2c). Quantitative analysis demonstrated significant astrocytic activation in the subiculum of fully kindled mice, as compared to the sham group (Figure 2d). To dissect the functional consequences of this astrocytic activation, we performed fiber photometry during kindling‐induced seizures (Figure 2e) and found that astrocytes, virally infected to express GCaMP6f, exhibited pronounced Ca^2+^ transients, which coincide with a burst of electrical activity recorded in the EEG (Figure 2g–i). One week later, we performed a fear conditioning test. Mice in both groups showed freezing behavior during the early consolidation (Figure 2j–l) and contextual recall (Figure 2m–o) associated with changes in astrocytic Ca^2+^ activity, which declined at the initiation of freezing (Figure 2k,n) and rose at the termination of freezing (Figure 2l,o). Kindled epileptic mice showed higher peak amplitudes of Ca^2+^ responses in the pre‐freezing epoch (measured at the initiation of freezing behavior and representing approximately the apex of Ca^2+^ response) when compared to sham controls (Figure 2k,n). This significant pre‐freezing activation in kindled mice may suggest that subicular astrocytes are excessively engaged in conditioned fear responses under a pathological hyperactivity state.

### 2.2 Suppression of Astrocyte Ca2+ Activity Reverses Seizure‐Induced Cognitive Impairment

**Figure 3:**
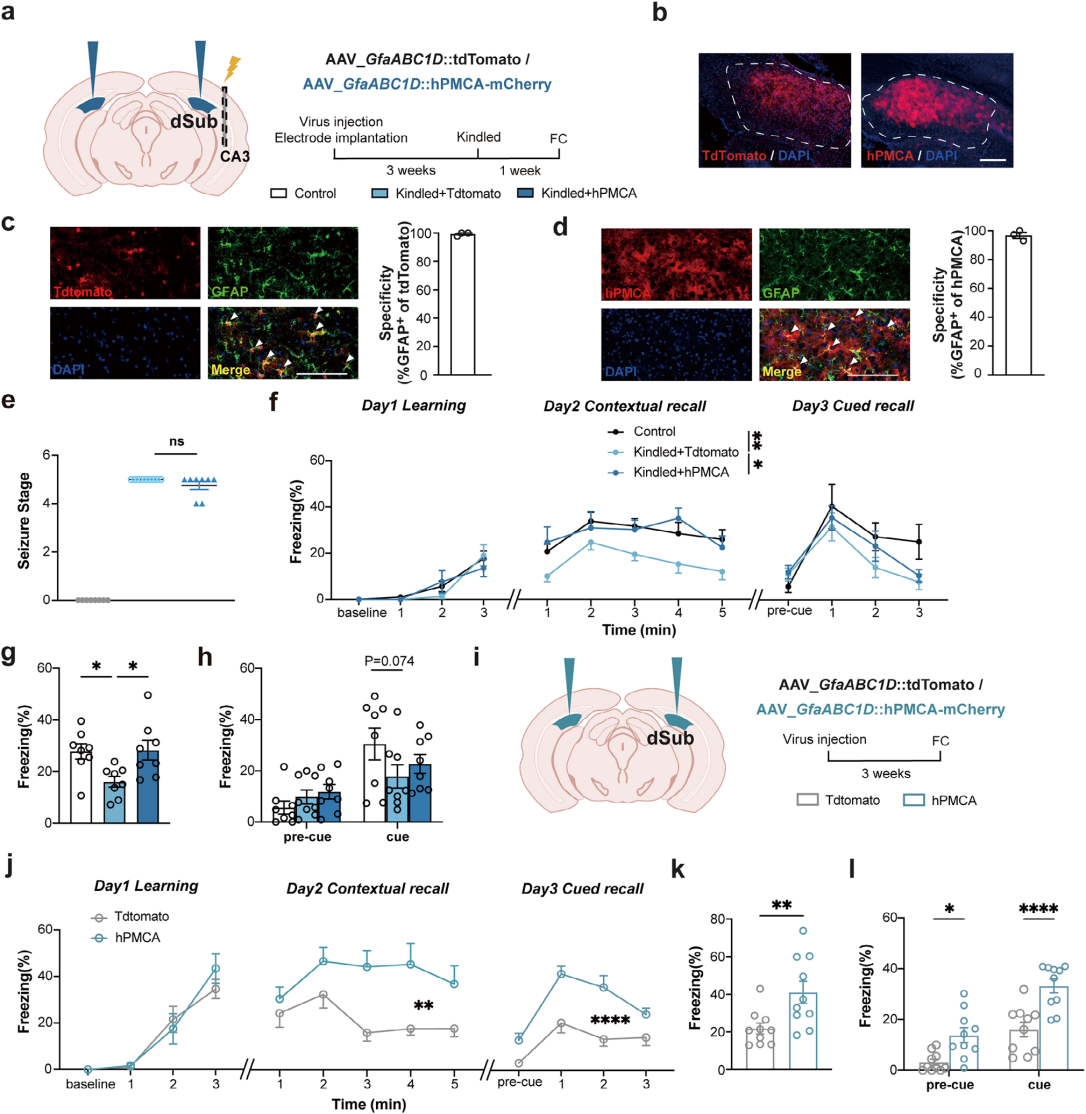
Suppression of astrocyte Ca^2+^ activity reverses seizure‐induced cognitive decline. a) Left, schematic of AAV5_*GfaABC1D*::hPMCA2w/b‐mCherry or AAV2/5_*GfaABC1D*::tdTomato (control virus) bilateral injection in dorsal subiculum and electrode implantation in the right ventral CA3 region. The control group has not received a viral injection, while electrodes were implanted, but no stimulus was provided. Right, schematic of the experiment. FC, fear conditioning. b) Representative images showing viral expression of tdTomato (left, red) hPMCA2w/b‐mCherry (right, red) with cell nuclei counterstained with DAPI (blue) in the dSub (dashed contour). Scale bar: 200 µm. c, d) Images: Immunofluorescence staining of GFAP and tdTomato (c) or hPMCA‐mCherry fluorescence (d); cell nuclei we stained using DAPI. Graphs: Co‐staining analysis demonstrates that both types of viruses had high specificity for astrocytes. Scale bar: 100 µm, n=3. e) Seizure stage of three groups (left‐ right: control, Kindled + tdTomato, and Kindled + hPMCA‐mCherry) (^**^P<0.01, ^****^P<0.0001, Brown‐Forsythe ANOVA test with Dunnett's T3 multiple comparisons test). f) The curve of freezing levels during the fear conditioning and memory retrieval (^*^P<0.05, ^**^P<0.01, two‐way ANOVA test with Tukey's multiple comparisons test). g) Percentage of freezing in contextual fear memory tested on Day 2 (^*^P<0.05, Brown‐Forsythe ANOVA test with Dunnett's T3 multiple comparisons test). h) Percentage of freezing in cued fear memory tested on Day 3 during memory acquisition (pre‐cue) or recall (cue), n=8 for all groups. i) Left, schematic of AAV5_*GfaABC1D*::mCherry‐hPMCA2w/b or AAV2/5_*GfaABC1D*::tdTomato (Control virus) bilateral injection in dSub. Right, schematic of the experiment. j) The curve of freezing level during the 3 day fear‐conditioning test (^**^P<0.01, ^****^P<0.0001, two‐way ANOVA test). FC as in a. k) Percentage of freezing in contextual fear memory tested on the second day (^**^P<0.01, unpaired *t*‐test). l) Percentage of freezing in cued fear memory tested on Day 3 (^*^P<0.05, ^****^P<0.0001, two‐way ANOVA test with Šídák's multiple comparisons test), n=11 for tdTomato group, n=10 for hPMCA group. Data are presented as mean ± SEM.

To establish the causal link between seizure‐induced astrocytic hyperactivity and fear memory dysfunction, we employed viral‐mediated approaches to specifically inhibit intracellular Ca^2+^ levels in subicular astrocytes. Firstly, we injected the virus, encoding human plasma membrane Ca^2+^‐ATPase isoform 2 splice variant w/b (hPMCA2w/b) appended at its Cs terminus with the red fluorescent protein mCherry; the expression of this chimeric protein was driven by the *GfaABC1D* promoter. The rationale for the expression of hPMCAw/b is to attenuate astrocytic Ca^2+^ activity, and implement the above kindling model (**Figure** **3**a). HPMCA2w/b efficiently transports intracellular Ca^2+^ to the extracellular space and decreases the amplitude and duration of both spontaneous and GPCR‐mediated Ca^2+^ signals in astrocytes.^[^ ^23^, ^24^ ^]^ In control, we used the virus encoding red fluorescent protein tdTomato, the expression of which is driven by the astrocytic specific *GfaABC1D* promoter. Both viruses expressed well and had high specificity to astrocytes as per colocalization of astrocytic marker GFAP with tdTomato (99.3%) or hPMCA2w/b‐mCherry (96.8%) (Figure 3b–d). Accordingly, we evaluated the efficiency of hPMCA2w/b on astrocytic Ca^2^⁺ reduction. We recorded dSub astrocytic Ca^2+^ signaling using mice virally co‐expressed GCaMP6f and hPMCA2w/b (Control: mCherry) (Figure S1a, b, Supporting Information). Astrocyte intracellular Ca^2+^ dynamics were recorded by fiber photometry and analyzed during a 3 day fear conditioning test. We observed that the astrocytic Ca^2+^ response during foot shock and freezing behavior significantly diminished in the hPMCA2w/b group when compared to mCherry mice (Figure S1c–k, Supporting Information). As above, we implanted individual dual EEG and stimulation electrodes into the right ventral CA3. These two groups of mice were stimulated with electrodes until fully kindled. The additional control group received no viruses, and mice were not stimulated, albeit electrodes were implanted. The seizure severity/stage was similar between the two groups of kindled animals, while the unstimulated control group predictably had not developed seizures (Figure 3e).

Previous studies have demonstrated that kindling of limbic epileptic foci (e.g., basolateral amygdala or the dorsal hippocampus) impairs hippocampal‐dependent fear conditioning.^[^ ^25^, ^26^, ^27^ ^]^ Under our experimental paradigm, kindled epileptic mice, which had virally mediated tdTomato expression, showed preserved fear acquisition capabilities, while contextual memory was significantly impaired, and the cued memory decline trend approached statistical significance when compared to control mice that were not kindled (Figure 3f–h). However, hPMCA2w/b‐mCherry expression in astrocytes of kindled mice sufficiently reversed this memory impairment (Figure 3f,g). Furthermore, hPMCA2w/b‐mCherry expression in kindled mice showed no notable effect on seizure progression susceptibility, suggesting astrocytes are selectively engaged in cognitive modulation rather than epileptogenesis (Figure S2a–e, Supporting Information).

To further elucidate whether astroctic Ca^2+^ activity is necessary for fear memory, we directly reduced astrocytic Ca^2+^ activity using virally expressed hPMCA2w/b‐mCherry in physiological conditions (Figure 3i); a control group of mice virally expressed tdTomato in astrocytes. The results revealed that reducing astrocytic Ca^2+^ activity did not affect fear acquisition, while an increase in contextual and cued fear memory was observed (Figure 3j,k). Mice with hPMCA2 w/b‐mCherry expression in astrocytes had significantly higher freezing in the contextual fear memory test on day 2. These mice also demonstrated a significant increase in freezing levels during the pre‐cue and cue periods, suggesting that mice with hPMCA2w/b expression in astrocytes may enhance fear generalization (Figure 3j,l).

In summary, physiological maintenance of moderately low astrocytic Ca^2^⁺ signaling in the subiculum enhances contextual fear memory. Seizure‐induced cognitive impairment may be linked to abnormally elevated Ca^2+^ activity in astrocytes, while reducing Ca^2+^ activity of these glial cells ameliorates these detrimental effects.

### 2.3 Activation of the Gq Signaling Pathway in Subicular Astrocytes Replicates Cognitive Impairment

**Figure 4:**
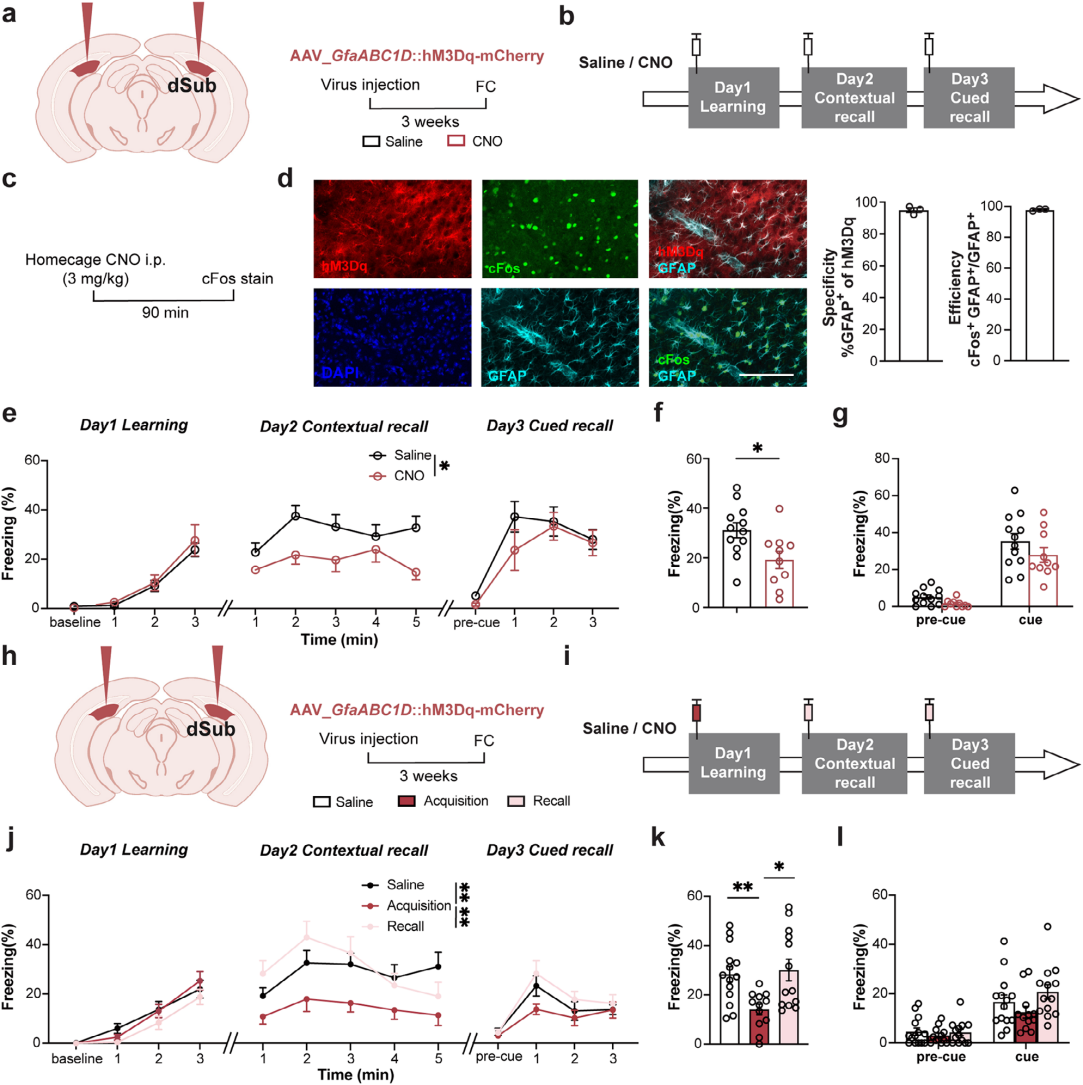
Activation of Gq signaling in subicular astrocytes directly induces cognitive impairment. a) Left, schematic of rAAV2/5_*GfaABC1D*::hM3Dq‐mCherry bilateral injection in dorsal subicula (dSub); Right, schematic of the experiment. Fc, fear conditioning. b) Schematic of the 3 day fear‐conditioning test with Gq activation. CNO, clozapine N‐oxide, an hM3Dq agonist. Saline is used as a control. c) Schematic of astrocyte activation (assessed by cFos+ stain) by 3 mg kg^−1^ CNO in the home cage. d) HM3Dq–mCherry fluorescence, along with immunochemistry co‐staining of GFAP and cFos in the CNO group. Cell nuclei are counterstained with DAPI. Graph‐left. High specificity of viral delivery to astrocytes, as per colocalization of hM3Dq‐mCherry and immunostaining for GFAP (left). Graph‐right. CNO activation of hM3Dq leads to astrocyte activation, as almost the entire astrocyte population (GFAP+) is cFos‐positive. Scale bar: 100 µm, n=3. e) The curve of freezing level during the 3 day fear‐conditioning test with (CNO) or without (saline) the hM3Dq/Gq pathway activation in astrocytes (^*^P<0.05, two‐way ANOVA test). hM3Dq activation significantly reduces the contextual recall, tested on day 2. f) Percentage of freezing in contextual fear memory tested on the second day (^*^P<0.05, unpaired *t*‐test). g) Percentage of freezing in cued fear memory tested on Day 3 (^****^P<0.0001, two‐way ANOVA test), n=12 for Saline group, n=10 for CNO group. h) Left, schematic of rAAV2/5_*GfaABC1D*:hM3Dq‐mCherry bilateral injection in dSub; Right, time‐line of the experiment; FC as in a. i) Schematic of the 3 day fear conditioning test with astrocyte Gq activation affecting memory acquisition and recalls (red and pink syringes, respectively). j) Curves of freezing levels during the 3 day fear‐conditioning test with astrocytic Gq activation during memory acquisition or memory recall; saline, control group is shown in black points (^**^P<0.01, two‐way ANOVA test with Tukey's multiple comparisons test). k) Percentage of freezing in contextual fear memory tested on Day 2 (^*^P<0.05, ^**^P<0.01, Brown‐Forsythe ANOVA test with Dunnett's T3 multiple comparisons test). l) Percentage of freezing in cued fear memory tested on the third day (^*^P<0.05, ^**^P<0.01, ^***^P<0.001, two‐way ANOVA test with Šídák's multiple comparisons test), n=14 for Saline group (open bars), n=12 for Acquisition group, n=13 for Recall group. Data are presented as mean ± SEM.

Next, we increased subicular astrocytic Ca^2+^ activity to mimic seizure‐induced astrocytic hyperactivation. Previous studies have demonstrated that the activation of the Gq pathway in astrocytes via hM3Dq, a modified form of the human M3 muscarinic that can be activated by the ligand clozapine N‐oxide (CNO), results in an increase in astrocytic Ca^2+^ levels.^[^ ^23^ ^]^ We bilaterally injected *GfaABC1D*::hM3D(Gq)‐mCherry virus into the dorsal subiculum (**Figure** **4**a). We administered the CNO (3 mg kg^−1^) via intraperitoneal injection to evaluate its sufficiency for chemogenetic activation of hM3Dq in astrocytes (Figure 4c). As expected, CNO dramatically increased c‐Fos expression in almost the entire astrocyte population (97.9 %), and this virus demonstrated high specificity for astrocytes (94.9%) (Figure 4d). We recorded astrocytic Ca^2+^ signaling using virally co‐expressed GCaMP6f and hM3Dq‐mCherry in these cells and conducted fiber photometry (Figure S3a, b, Supporting Information). We observed that the astrocytic Ca^2+^ response increased by hM3Dq activation (Figure S3c, d, Supporting Information). Consistent with our prior observations with hPMCA2w/b, chemogenetic activation via hM3Dq demonstrated no significant impact on seizure susceptibility (Figure S4a–e, Supporting Information), suggesting astrocytic regulation does not affect epileptogenesis. Additionally, hM3Dq activation of subicular astrocytes has no impact on locomotor or anxiety‐like behavior (Figure S5a–c, Supporting Information). Subsequently, we used a full‐time modulation approach, which incorporated the administration of CNO/saline 30 min prior to both the learning and memory retrieval phases (Figure 4b). We observed no notable difference in freezing proportion during fear acquisition/learning (day 1) or cued memory (day 3) tests, whereas contextual fear memory (day 2) exhibited a significant decrease (Figure 4e–g). The results suggest that activating subicular astrocytes via the Gq pathway leads to contextual fear memory impairment.

To delineate the phase of cognitive impairment to which astrocytic hyperactivity contributes, we selectively modulated the memory acquisition and retrieval phases. Specifically, we timed CNO administration 30 min before distinct memory processing stages: the learning phase (aligned with consolidation) and the retrieval phases (Figure 4h,i). Experimental results demonstrated that the acute CNO administration during the learning phase (pre‐consolidation window) fully mimicked the effects of the full‐time modulation, causing a marked attenuation of contextual fear memory (Figure 4j–l). In contrast, targeted astrocytic manipulation during the memory recall phase failed to alter fear memory expression, as evidenced by unchanged freezing behavior (Figure 4j–l). These findings establish that the subicular astrocytic activity exerts its critical influence specifically during the early consolidation phase of the contextual fear memory formation.

In summary, learning‐associated astrocytic hyperactivity specifically contributes to cognitive impairment by disrupting the memory acquisition phase (early consolidation) rather than the memory retrieval phases.

### 2.4 Subicular Astrocytes Activation Affects Local Neuronal Excitability for Fear Processing

**Figure 5:**
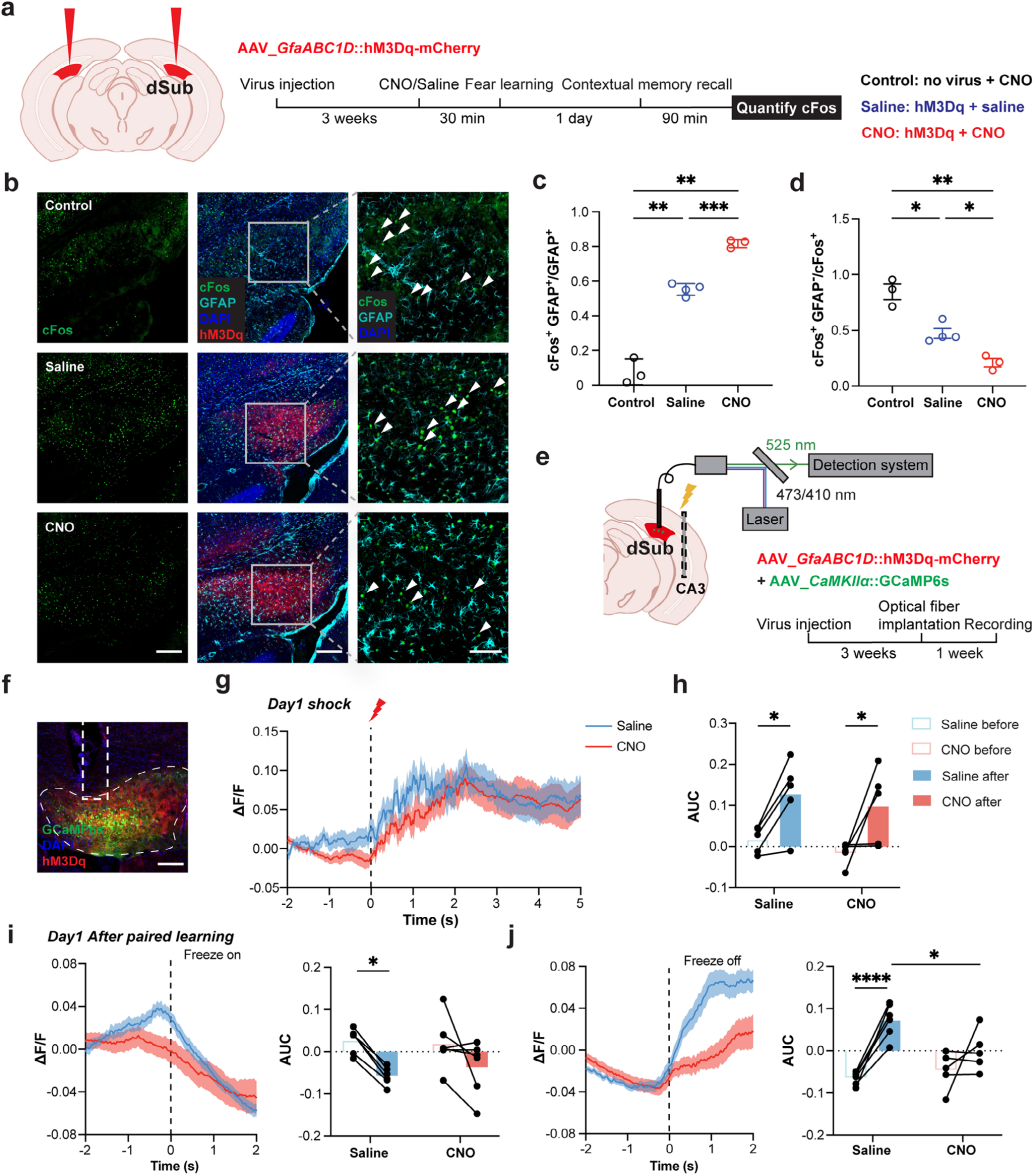
Activation of Gq signaling in subicular astrocytes decreases local neuronal response in fear memory. a) Left, schematic of rAAV2/5_*GfaABC1D*::hM3Dq‐mCherry bilateral injection in dorsal subiculum (dSub); Right, Timeline of the astrocyte Gq activation experiment with cFos quantification. Saline or CNO (agonist) was administered to mice injected with rAAV2/5_*GfaABC1D*::hM3Dq‐mCherry; CNO was also injected in control mice, which were virally transduced. 30 min later, mice underwent fear conditioning. 90 min after the contextual memory recall, brains were removed, sliced, and stained for cFos. b) Representative fluorescence images of hM3Dq‐mCherry (red) along with immunofluorescence co‐staining of cFos (green) and GFAP (teal). Cell nuclei are counterstained with DAPI (blue). Rectangles in the mid columns are shown as close‐up images in the right column, but devoid of the hM3Dq‐mCherry signal. Scale bar: 200 µm (Left and mid columns), 50 µm (right column). Arrowheads in the right column indicate the individual cFos without co‐label GFAP. c) Quantification of the proportion of cFos^+^ GFAP^+^ astrocytes (^**^P<0.01, ^***^P<0.001, Brown‐Forsythe ANOVA test with Dunnett's T3 multiple comparisons test). d) Quantification of the proportion of cFos^+^ cells in other GFAP^−^ neural cells (^*^P<0.05, ^**^P<0.01, Brown‐Forsythe ANOVA test with Dunnett's T3 multiple comparisons test), n=3 for Control group, n=4 for Saline group, n=3 for CNO group. e) Schematic of the astrocytic Gq activation experiment with cytosolic Ca^2+^ recordings from CaMKIIα^+^ neurons. Mice were injected with rAAV2/5_*GfaABC1D*::hM3Dq‐mCherry and rAAV2/R_*CaMKIIα*::GCaMP6s in the right dSub. Three weeks later, an optic fiber was implanted above the dSub along with a dual electrode in the CA3 region. After the one‐week recovery, mice underwent fear conditioning while recording neuronal cytosolic Ca^2+^ levels using photometry. f) Representative fluorescence image of GCaMP6s (green), DAPI (blue), and hM3Dq‐mCherrry (red) in the dSub (dashed contour). Electrode placement is shown by the vertical dashed rectangle. Scale bar: 200 µm. g) Average Ca^2+^ photometry signal from neurons, in mice receiving CNO or saline, during day 1 foot shocks, the timing of which is indicated by the vertical line and electrical bolt. h) Quantification of neuronal Ca^2+^ signal as area under the curve (AUC) 2 s before the shock and 2 s after the shock (^*^P<0.05, two‐way ANOVA test with Šídák's multiple comparisons test). i) Left, Average neuronal Ca^2+^ photometry signal during the Day1 freezing initiation, indicated by the vertical dashed line. Right, Quantification of such AUC 2 s before and 2 s after the freezing initiation (^*^P<0.05, two‐way ANOVA test with Šídák's multiple comparisons test). j) Left, Average neuronal Ca^2+^ photometry signal during the Day 1 freezing termination. Right, Quantification of such AUC 2 s before and 2 s after freezing termination (^*^P<0.05, ^****^P<0.0001, two‐way ANOVA test with Šídák's multiple comparisons test, n=6 for Saline group, n=5 for CNO group. Data are presented as mean ± SEM.

Given that subicular neurons are involved in contextual fear memory retrieval,^[^ ^14^ ^]^ we sought to investigate whether surrounding astrocytes directly influence these neuronal ensembles during fear processing. As the first step to evaluate the effect of astrocytes on local neuronal excitability, we characterized cFos^+^ labelling of neural cells in the subiculum 90 min after contextual memory retrieval (**Figure** **5**a). The animals used here were bilaterally injected with *GfaABC1D*::hM3Dq‐mCherry virus into the dorsal subiculum and systemically administered either saline or CNO (saline and CNO groups, respectively). We also used the control group of animals that were spared from viral injections, but administered with CNO. A statistical analysis of astrocyte activation revealed that the CNO group had a significantly higher proportion of cFos^+^ astrocytes compared to the saline group, suggesting effective and specific astrocyte activation by CNO that persisted after contextual retrieval (Figure 5b, c). Additionally, we found that the saline group showed a higher number of cFos^+^ astrocytes than the control group (Figure 5c), potentially due to the expression of the mCherry fluorescent protein itself, which made subicular astrocytes more susceptible to activation.^[^ ^28^ ^]^ The CNO group had the highest proportion of cFos^+^ astrocytes, dwarfing both control and saline groups, consistent with the premise that Gq activation of astrocytes leads to cFos^+^ expression. It should be noted that in control group (receiving CNO, but no viral delivery), the non‐astrocyte‐derived c‐Fos^+^ stain was prominent and the most preponderant of the three groups. However, the proportion of non‐astrocyte‐derived c‐Fos^+^ decreased following astrocytic activation, suggesting a reduction in the number of subicular neurons involved in fear memory (Figure 5d).

Conversely, mice were also sacrificed 90 min after contextual retrieval and cFos staining in the hPMCA mice was performed (Figure S6a, b, Supporting Information). Consistent with previous results, mice in the hPMCA group exhibited a significantly higher contextual fear memory level compared to control groups (both tdTomato and mCherry) (Figure S6a–d, Supporting Information). cFos expression was significantly higher in the hPMCA group compared to the control group, while cFos expression in astrocytes was significantly lower in the hPMCA group compared to the control group (Figure S6e–g, Supporting Information). These results indicate that decreasing subicular astrocytes' Ca^2+^ activity is sufficient to reduce the expression of cFos in astrocytes, while it increases the total cFos expression mainly in subicular neurons.

Next, we utilized in vivo fiber photometry to monitor real‐time Ca^2+^ signals in subicular CaMKIIα^+^ neurons, simultaneously with activation of surrounding astrocytes. We bilaterally injected a cocktail of AAV‐*GfaABC1D*::hM3D(Gq)‐mCherry and AAV‐*CaMKIIα*::GCaMP6s viruses into the dorsal subiculum. Three weeks after virus expression, optical fibers were implanted into the CA3 region, and Ca^2+^ signal recording commenced one week later (Figure 5e, f). Animals were split into two groups, one receiving CNO and the other saline. Initially, both groups exhibited similar responses to electrical stimulation (footshock), showing heightened post‐shock Ca^2+^dynamics (Figure 5g, h). Subsequently, we analyzed the Ca^2+^ dynamics at the initiation and termination of freezing behavior. The saline group showed significant reduction and increase in post‐shock Ca^2+^ dynamics during the day 1 freezing initiation and freezing termination, respectively (Figure 5i,j). Upon astrocytes Gq activation (CNO group), these Ca^2+^ dynamics during memory consolidation were suppressed (Figure 5i,j), showing no significance between signals prior to and after the shock. Similar statistically insignificant changes in Ca^2+^ dynamics were observed during contextual (Figure S7a, b, Supporting Information) and cued (Figure S7c, d, Supporting Information) memory retrievals. Collectively, these results suggest that astrocytic activation reduces the local CaMKIIα^+^ neuron excitability in response to freezing behavior.

To investigate whether a similar suppression of CaMKIIα^+^ neurons in the subiculum also occurs in epileptic mice, we bilaterally injected the AAV‐*CaMKIIα*::GCaMP6s virus into the dorsal subiculum and implanted optical fibers and electrodes into the dorsal subiculum and CA3, respectively (Figure S8a, b, Supporting Information). Once the mice were fully kindled (following three weeks of viral expression), fear conditioning was performed 1 week later. We observed that the neuronal Ca^2+^ response during foot shock was comparable between the 2 groups (Figure S8c, d, Supporting Information), while the CaMKIIα^+^ neuronal Ca^2+^ response during freezing behavior was significantly diminished in the Kindled group during consolidation (Figure S8e–h, Supporting Information).

To directly manipulate neuronal activity, we expressed hM3Dq in subicular CaMKIIα^+^ neurons (Figure S9a, Supporting Information). We confirmed that 96.1% of the hM3Dq‐mCherry^+^ neurons were CaMKIIα^+^ (Figure S9b, Supporting Information). Three weeks later, we injected CNO or saline 30 min before the memory retrieval (Figure S9c, Supporting Information). During the 3 day conditioned fear memory test, mice in the CNO group exhibited a significantly higher freezing curve, showing the enhancement of contextual fear memory (Figure S9d–f, Supporting Information). Additionally, we quantified cFos expression following the contextual memory retrieval and found higher cFos levels in the CNO group (Figure S9g, h, Supporting Information). These results support that activation of CaMKIIα^+^ neurons in the dorsal subiculum enhances contextual memory retrieval.

Together, selective activation of subicular astrocytes significantly inhibited the engagement of local CaMKIIα^+^ neurons in contextual memory, indicating a complex interaction between neurons and astrocytes in fear memory processing.

### 2.5 Subicular Astrocytes Modulate Fear Memory via Astrocyte‐Neuron Adenosine‐Mediated Signaling

**Figure 6:**
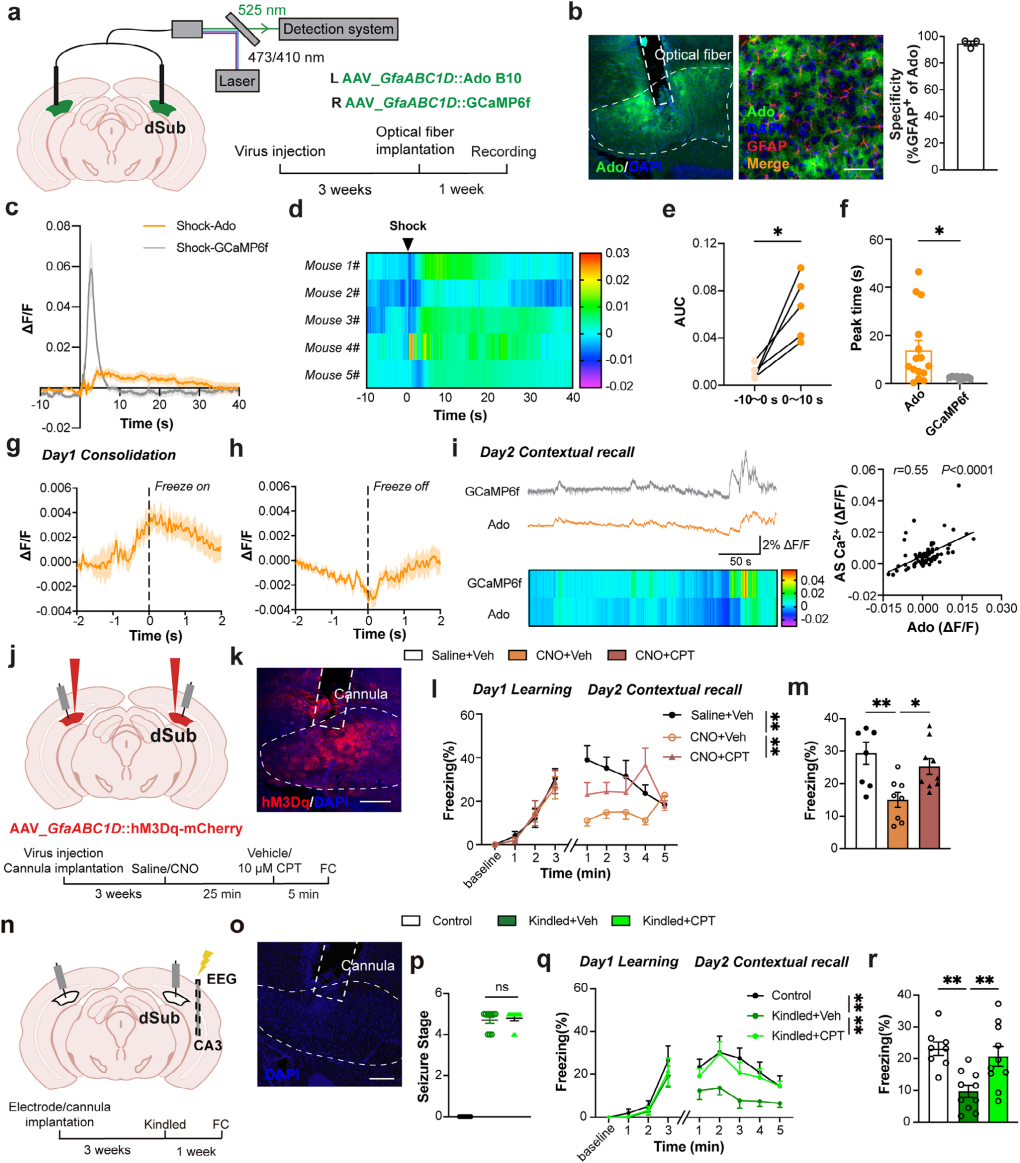
Subicular astrocytic activation drives cognitive impairment via the adenosine signaling pathway. a) Left, schematic of rAAV2/9_*GfaABC1D*::Ado_B10 injection in the left (L; ipsilateral) dorsal subiculum (dSub), and AAV5_*GfaABC1D*::GCaMP6f injection in the right (R; contralateral) dSub. Right, schematic of the experiment. b) Images: Left, a typical diagram image of dSub (dashed contour) expressing the extracellular adenosine (Ado; green) sensor; cell nuclei were counterstained with DAPI. The optic fiber location is indicated by the dashed vertical rectangle. Scale bar: 200 µm. Right, immunofluorescence of GFAP (red) with Ado (green) and DAPI (blue) fluorescence. Graph: Co‐localization analysis of the dSub shows astrocyte‐specific expression of Ado as this signal co‐localizes almost to its entirety with that of GFAP^+^ cells, i.e., astrocytes. Scale bar: 100 µm, n=3. c) Recordings of extracellular adenosine levels and astrocyte cytosolic Ca^2+^ dynamics following foot shocks (delivered at time = 0), n=5, 3 trials per mouse. d) Heatmap of extracellular adenosine levels. Vertical bar (right) displays a pseudo color representation of ΔF/F ranging from ‐0.02 to 0.03. e) Quantification of extracellular adenosine levels reported as the area under the curve (AUC) 10 s before and 10 s after shock (^*^P<0.05, paired t test). f) Quantification of time to reach the peak for extracellular adenosine and cytosolic Ca^2+^ signals (^*^P<0.05, unpaired t test), n=5 for Ado group, n=3 for GCaMP6f group, 3 trials per mouse. g, h) Quantification of extracellular adenosine dynamics (ΔF/F) at the initiation or termination of freezing behavior during the Day1 post‐learning period, with each event occurring at the respective vertical dashed line (time=0). 11 trials from 4 mice. i) Left, representative ΔF/F traces and heatmaps of astrocytes' Ca^2+^ and extracellular adenosine signals recorded simultaneously, albeit in contralateral dSub. Vertical bar displays a pseudo color representation of ΔF/F with a marked range from ‐0.02 to 0.04. Right, Positive linear correlation between extracellular Ado and astrocyte Ca^2+^ signals. j) Top, schematic of bilateral rAAV2/5_*GfaABC1D*::hM3Dq‐mCherry injection in dSub and bilateral cannula implantation (syringe symbol) above dSub; Bottom, Experimental schematics. FC, fear conditioning; CNO, clozapine N‐oxide; CPT, 8‐cyclopentyltheophylline. k) A representative image of a dSub (dashed contour) expressing hM3Dq‐mCherry in astrocytes (red) with cell nuclei counterstained with DAPI (blue). The cannula placement is indicated by a dashed rectangle. Scale bar: 200 µm. l) The curve of freezing level during the fear conditioning and contextual memory recall; Veh, vehicle. (^*^P<0.05, ^**^P<0.01, two‐way ANOVA test with Šídák's multiple comparisons test). m) Percentage of freezing in contextual fear memory tested on Day 2 during memory acquisition and contextual memory recall (^*^P<0.05, Brown‐Forsythe ANOVA test with Dunnett's T3 multiple comparisons test), n=7 for Saline+Veh group, n=8 for CNO+Veh group, n=9 for CNO+CPT group. n) Top, schematics of bilateral cannula implantation above dSub and electrode implantation in the right CA3; Bottom, schematic of the experiment. FC, fear conditioning. o) A representative image of a dorsal subiculum (dashed contour) with DAPI‐stained cell nuclei (blue). The cannula placement is indicated by a dashed rectangle. Scale bar: 200 µm. p) Seizure stage of three groups (^***^P<0.001, ^****^P<0.0001, Brown‐Forsythe ANOVA test with Dunnett's T3 multiple comparisons test). q) The curve of freezing level during the fear conditioning and memory retrieval. (^**^P<0.01, two‐way ANOVA test with Tukey's multiple comparisons test). r) Percentage of freezing in contextual fear memory tested on day 2 (^**^P<0.01, Brown‐Forsythe ANOVA test with Dunnett's T3 multiple comparisons test), n=8 for Control (unkindled and no Veh/CPT) group, n=10 for Kindled+Veh group, n=10 for Kindled+CPT group. Data are presented as mean ± SEM.

Previous studies demonstrated that astrocytes regulate the excitability of local neurons by releasing several gliotransmitters.^[^ ^29^, ^30^ ^]^ To further investigate which astrocyte‐derived gliotransmitters are involved in the regulation of fear memory, we focused on adenosine and glutamate, major inhibitory and excitatory hippocampal gliotransmitters, respectively. Nonetheless, we virally expressed extracellular adenosine (AAV_*GfaABC1D*::Ado_B10) or glutamate sensor (AAV_*GfaABC1D*::iGluSnFR(A184S)) at astrocytes' plasmalemma in one subiculum, while astrocytes in the collateral subiculum expressed the cytosolic Ca^2+^ sensor GCaMP6f (AAV_*GfaABC1D*::GCaMP6f); ipsilateral expression of transmitter and Ca^2+^ sensors is not warranted due to gross overlaps in their blue excitation/ green emission spectra. After three weeks of viral expression, optic fibers were bilaterally implanted in the dorsal subiculum to simultaneously monitor contralateral dynamics in extracellular gliotransmitter (either adenosine or glutamate) and astrocytic Ca^2+^ levels (**Figure** **6**a; Figure S9a, Supporting Information). Post‐validation confirmed the position of the optic fiber, while immunohistochemical co‐staining with GFAP demonstrated high astrocytic specificity for viral delivery (Figure 6b; Figure S9b, Supporting Information). Our results showed that adenosine release by subicular astrocytes increased after foot shock, with a sustained elevation for ≈30 s (Figure 6c–e). In contrast, there was no significant change in glutamate levels, albeit a simultaneous contralateral shock‐elicited Ca^2+^transient was readily recorded (Figure S10a–g, Supporting Information). Notably, the peak time of adenosine release was significantly (tens of seconds) delayed compared to the peak time of astrocytic Ca^2+^ signal (Figure 6f). This finding suggests that adenosine may be released through the calcium‐dependent exocytotic release pathway. These results suggest that during foot shock, subicular astrocytes' Ca^2+^ activity gets elevated, accompanied by a long‐lasting astrocyte‐derived adenosine release.

Similar to cytosolic Ca^2+^ activity, adenosine levels began to increase before the onset of freezing behavior, peaked at the start of freezing, and subsequently declined to reach the lowest point at the end of freezing, followed by a rebound in adenosine signaling (Figure 6g,h). There were apparent correlative dynamics between the cytosolic Ca^2+^/GCaMP6f signal and extracellular adenosine levels/Ado signal during the contextual fear memory retrieval (Figure 6i). To further validate that adenosine is released via a calcium‐dependent exocytotic mechanism, we recorded astrocytic extracellular adenosine response using virally co‐expressed Ado probe and hPMCA2w/b (Control: mCherry) in these cells (Figure S11a, b, Supporting Information). By using fiber photometry, the results showed that the astrocytic extracellular adenosine response during foot shock and freezing behavior was significantly diminished in the hPMCA2w/b group when compared to mCherry mice (Figure S11c–f, Supporting Information). These findings demonstrate that astrocytic adenosine release coincided with intracellular Ca^2^⁺ signaling and that subicular astrocytes may regulate fear memory through adenosine‐mediated pathways.

The primary receptor for adenosine in the hippocampus is the adenosine A_1_ receptor (A_1_R).^[^ ^31^ ^]^ The activation of both presynaptic and postsynaptic A1Rs influences neuronal excitability and synaptic transmission.^[^ ^32^, ^33^ ^]^ To assess the involvement of adenosine signaling in fear memory regulation, we next performed pharmacological intervention by administering the A_1_R antagonist 8‐cyclopentyltheophylline (CPT; 10 µmol concentration) in the dorsal subiculum using bilateral cannulas. In these experiments, we bilaterally and virally (AAV_*GfaABC1D*::hM3Dq‐mCherry) expressed hM3Dq‐mCherry in subicular astrocytes. We also bilaterally implanted cannulas into the dorsal subiculum. We conducted behavioral experiments three weeks later (Figure 6j,k). We administered CNO to activate astrocytes 30 min prior to fear conditioning, followed by CPT delivery through the cannulas to block A_1_Rs in subiculum 5 min before learning. The results demonstrated that CNO‐hM3Dq‐activatied significantly reduced contextual fear memory/recall when compared to the saline‐hM3Dq group, while CPT reversed the CNO‐hM3Dq‐mediated impairment in the contextual recall (Figure 6l,m). We further assessed the utilization of this pathway in pathological, epileptic conditions. We implanted cannulas bilaterally into the dorsal subiculum and a stimulation electrode in the CA3 region, the latter used to conduct the kindling model (Figure 6n,o). The seizure severity was similar between the kindled groups (Figure 6p). Behavioral results showed that administration of CPT was sufficient to reverse the decline in freezing levels seen in epileptic mice (Figure 6q,r). However, no significant change in cued memory was detected in the kindled group (Figure S12a–c, Supporting Information).

Taken together, our findings establish that subicular astrocytes regulate fear memory via adenosine signaling, whereby an activity‐dependent adenosine release reduces neuronal excitability through the activation of A1Rs. Notably, under epileptic conditions, pathological activation of these astrocytes drives cognitive deficits through A1R‐mediated tonic inhibition of fear‐encoding neurons in the subiculum.

## 3 Discussion

In recent years, an increasing number of studies have demonstrated the involvement of astrocytes in cognitive disorders, providing a reliable target for seizure‐related cognitive impairment. Our present study specifically targeted the subiculum, a crucial hippocampal output region that is indispensable for the retrieval of contextual fear memory in epilepsy. We revealed three key findings: First, subicular astrocytes exhibit activity‐dependent engagement during fear memory acquisition and undergo pathological hyperactivation following seizures. Second, suppression of astrocytic Ca^2+^ signaling fully rescues seizure‐driven memory deficits, while selective Gq pathway activation in astrocytes before fear learning replicates this dysfunction. Third, mechanistic investigations show that seizure‐induced Ca^2+^ transients in subicular astrocytes trigger activity‐dependent adenosine release, which suppresses excitatory CaMKIIα^+^ neuronal activity through adenosine A1Rs. Collectively, these findings clarify the critical role and underlying mechanisms of subicular astrocytes in seizure‐related cognitive impairment, providing potential drug targets.

The subiculum, serving as the principal efferent hub of the hippocampus, exhibits marked subregion‐specific functional heterogeneity in its astrocytic regulatory dynamics. Real‐time monitoring of Ca^2+^ signals has become indispensable for elucidating the role of astrocytes in fear conditioning.^[^ ^34^, ^35^ ^]^ Our results showed that astrocytes exhibit an immediate and transient cytosol Ca^2+^ response to the foot shock, aligning with recent studies reporting similar astrocyte responses in the basolateral amygdala (BLA).^[^ ^35^ ^]^ Interestingly, we observed a significant increase in Ca^2^⁺ activity (when mice first enter the fear conditioning chamber) related to the active state after learning. As a mere active state in a novel environment is insufficient to elicit significant Ca^2^⁺ activity, it appears that Ca^2^⁺ activity in astrocytes primarily depends on significant stimuli during paired learning. In kindled epileptic mice, we observed increased astrocyte Ca^2+^ activity preceding freezing onset in the kindled group, which may indicate either that pathologically enhanced astrocyte activity directly induces the freezing state or that it correlates with local neuronal activation prior to freezing. Moreover, the activity increases following freezing cessation, which possibly indicates that astrocytes are involved in inhibiting fear and anxiety states,^[^ ^36^ ^]^ or it may correlate with the initiation of mouse movement.^[^ ^37^ ^]^ Bidirectional manipulation of subicular astrocytic Ca^2^⁺ dynamics established their causal modulatory effects on contextual fear memory under both physiological and pathological (epileptic hyperactivation) conditions. We found that reducing Ca^2+^ activity by hPMCA enhances fear memory. Similarly, regulating hippocampal CA1 astrocytes through hPMCA enhances contextual fear memory in mice.^[^ ^38^ ^]^ Previous research has demonstrated that the mechanism underlying hPMCA in astrocytes involves the reduction of tonic inhibition in striatal spiny neurons and the modulation of local microcircuits.^[^ ^23^ ^]^ In parallel, we utilized chemogenetics to activate the Gq‐coupled signaling pathway in subicular astrocytes, which led to ‌stage‐selective suppression‌ of contextual fear memory. Intriguingly, an earlier study found that activating the Gq signaling pathway in dorsal CA1 astrocytes facilitated long‐term potentiation and enhanced subsequent contextual fear memory retrieval.^[^ ^39^ ^]^ These apparently contradictory findings could arise from functional heterogeneity among hippocampal subregions.^[^ ^14^, ^40^ ^]^

Furthermore, our findings revealed that subicular astrocytes exhibit stage selectivity in fear memory. Activating astrocytes prior to memory acquisition, but not during retrieval, exerts an inhibitory effect on context fear memory. Consistent with this view, previous studies in other brain regions have shown that regulating astrocytes during memory retrieval does not affect the recall of recent or remote memories.^[^ ^39^, ^41^ ^]^ This leads us to hypothesize that astrocytes within various brain regions possess distinct characteristics in regulating fear memory. Here, the elevation of astrocytic Ca^2+^ activity in “specific fear contexts” following associative learning could represent an endogenous protective mechanism, acting as scavenger cells showing inhibition on later context fear memory. Increasing astrocytic Ca^2+^ activity during early consolidation inhibits the plasticity enhancement of subicular neuronal ensembles by releasing adenosine, thereby preventing memory allocation. While in later stages, astrocytes may be unable to exert additional influence on neuronal ensembles that have already transitioned into neuronal engram cells. Engram neurons within the subiculum are essential for the retrieval of contextual memories.^[^ ^14^ ^]^ We show that chemogenetic activation of subicular CaMKIIα^+^ neurons potentiates contextual fear memory, underscoring astrocytes as critical regulators of neuronal engram dynamics. Research elsewhere has reported that the increased Ca^2+^ activity following conditioned fear learning may be associated with memory formation, where astrocytes provide lactate to neurons to support memory encoding and consolidation.^[^ ^42^ ^]^ However, in our present research, hyperactivation of astrocytes may lead to the opposite effect. In vivo fiber photometry revealed tight coupling between subicular astrocytic Ca^2^⁺ transients and extracellular adenosine dynamics, establishing adenosine as the principal humoral factor mediating astrocyte‐dependent fear memory. Both A_1_R and A_2A_R have been implicated in learning and memory processes, the hippocampus exhibits high expression of A_1_R, which primarily serves to inhibit excitatory neurotransmission in the brain.^[^ ^43^, ^44^ ^]^ Pharmacological blockade of A_1_R significantly attenuates contextual fear memory, establishing A_1_R as a critical receptor. Recently, the concept of the learning‐associated astrocyte (LAA) ensemble has emerged.^[^ ^9^ ^]^ How these LAA are activated and how they interact with the neuronal ensemble in memory modulation remain to be elucidated.

In conclusion, our findings identify subicular astrocytes as critical modulators of seizure‐associated cognitive dysfunction through driving astrocytic Ca^2+^dependent adenosine signaling that disrupts neural circuit homeostasis through the activation of A_1_Rs. This highlights astrocyte‐targeted interventions as a therapeutic strategy for seizure‐related memory disorders.

## 4 Experimental Section

### Animals

Male mice at 7–14 weeks of age were used in this study, SPF grade, provided by the Zhejiang University Laboratory Animal Center (Hangzhou, China). For the housing environment, the animals were fed with water freely, with a daily 12 h light‐dark cycle (8:00‐20:00 light), and daily behavioral experiments were controlled between 9:00 and 18:00. Mice were raised and used according to the guidelines of the Animal Advisory Committee of Zhejiang University and the US NIH Guidelines for the Care and Use of Laboratory Animals. All procedures were approved by the Animal Advisory Committee of Zhejiang University. All surgeries were performed under sodium pentobarbital anesthesia, and every effort was made to minimize suffering. The approval number for animal experiments is No.16804.

### Stereotactic Virus Injection

Mice were anesthetized with pentobarbital sodium (60 mg kg^−1^, i.p.) and were mounted on a stereotaxic apparatus (J2844301, RWD Life Science Co., Ltd). A total volume of virus solution (50 nl) was injected into the dorsal subiculum bilaterally (AP: −3.4 mm; ML: ±2.0 mm; DV: −1.8 mm) with a 1‐µL (microliter) syringe (Gaoge, China) controlled by an injection pump (Micro 4, World Precision, USA) at 20 nl min^−1^. The injection needle was withdrawn 10–15 min after the infusion. The coordinates were measured from the Bregma, according to the atlas of Franklin and Paxinos.^[^ ^45^ ^]^ Behavioral tests were conducted at least 3 weeks following virus injection. The accurate position of injection sites was assessed in all animals post hoc by preparing sections (30 µm) containing the subiculum. Only the mice with the correct locations of viral expression were taken into analysis.

For selective manipulation of astrocytes or neurons, mice were injected with rAAV2/5_*GfaABC1D*::hM3Dq‐mCherry (BrainVTA Co. Ltd, diluted to 5.90×10^12^ viral genome (vg) ml^−1^), AAV5_*GfaABC1D*:: hPMCA2w/b‐mCherry (WZ biosciences Inc, diluted to 8.04×10^12^ vg ml^−1^), and rAAV2/R_*CaMKIIα*::hM3D(Gq)‐mCherry (BrainVTA Co. Ltd, diluted to 2.59×10^12^ vg ml^−1^). AAV2/5_*GfaABC1D*::tdTomato (WZ biosciences Inc., diluted to 4.72×10^11^ vg ml^−1^) and AAV2/5_*GfaABC1D*::mCherry (Taitool, diluted to 3 × 10^12^ vg ml^−1^) were used as corresponding control viruses.

For Ca^2+^ signaling and probe recording, mice were injected with AAV5_*GfaABC1D*::GCaMP6f (BrainVTA Co. Ltd, diluted to 2.21 × 10^12^ vg ml^−1^) or rAAV2/R_*CaMKIIα*::GCaMP6s (BrainVTA Co. Ltd, diluted to 4.59 × 10^12^ vg ml^−1^). For the assessment of extracellular glutamate or adenosine levels, rAAV2/9_*GfaABC1D*::iGluSnFR(A184S) (BrainVTA Co. Ltd, diluted to 5.28 × 10^12^ vg ml^−1^) virus or rAAV2/9_*GfaABC1D*::Ado_B10 (BrainVTA Co. Ltd, diluted to 3.24 × 10^12^ vg ml^−1^) was used, respectively.

### Stereotactic Surgeries

For fiber or cannula implantation, mice were anesthetized with pentobarbital sodium (60 mg kg^−1^, i.p.) and mounted on a stereotaxic apparatus (J2844301, RWD Life Science Co., Ltd). Optical fiber (300 µm in diameter, NA: 0.22/0.37, Inper, China) was stereotactically implanted into the dorsal subiculum (AP: −3.4 mm; ML: ±2.0 mm; DV: −1.75 mm) to enable fluorometric monitoring. Cannula (62003, RWD Life Science Co., Ltd) was stereotactically implanted into the dorsal subiculum (Tilt 15°, AP: −3.4 mm; ML: ±2.4 mm; DV: −1.20 mm) to enable drug injection. Optical fiber and cannulas were immobilized using dental cement. Three screws were placed over the skull to fix the dental cement.

For electrode implantation, twisted‐bifilar stainless electrodes (795500, 0.127 mm diameter, A.M Systems, USA) were implanted into the right ventral hippocampal CA3 (AP −2.9 mm; ML −3.2 mm; DV −3.2 mm) for both kindling stimulation and EEG monitoring. Then three screws were placed over the skull to fix the dental cement, two of which were placed over the motor cortex and cerebellum to serve as the ground and reference electrodes, respectively.

The experiments began after at least one week of recovery. Upon completion of behavioral experiments, the implantation positions of the optical fibers, cannula, or electrode were assessed in all mice. Only data from mice with correct implantation sites were used for the result analysis.

### Fear Conditioning

The fear memory test was performed similarly to our previous studies.^[^ ^46^ ^]^ Before the behavioral experiments, mice were placed in the testing room for at least 30 min to allow for environmental adaptation. To mitigate anxiety in mice due to contact with the experimenter, mice underwent 5 min handling for 3–4 consecutive days. The fear learning and retrieval tasks were carried out in a 15 cm × 15 cm × 20 cm chamber (Panlab Harvard Apparatus) placed inside a sound‐protected box. The experiment spanned 3 days. On day 1, the chamber was deodorized and sanitized with 75% ethanol, and mice were allowed to freely explore the chamber for 120 s. Then at 120, 180, and 240 s, a 30 s auditory stimulus (conditioned stimulus, 5000 Hz, 80–85 dB) with a footshock (unconditioned stimulus, 0.4 mA, 2 s) was delivered. Each footshock was followed by a 30 s interval. Mice remained in the chamber for an additional 60 s to consolidate the memory. On day 2, mice were returned to the chamber and allowed to freely explore for 5 min without any auditory or electrical stimuli. On day 3, the chamber was cleaned with 1% acetic acid instead of ethanol. Mice were placed in a cylindrical, opaque plastic tube (12 cm diameter) with a black‐and‐white checkered interior surface, allowing for free exploration for 2 min, followed by a 3 min auditory stimulus. The experiment was recorded by Freezeframe software (FREEZING, Panlab Harvard Apparatus, USA), which automatically analyzes the freezing behavior of mice. Freezing was defined as the absence of movement for at least 1 s, and the total duration of freezing bouts was quantified.

### Hippocampal Kindling Model

For the hippocampal kindling model, after 1 week of recovery, the EEG of the right ventral hippocampal CA3 was recorded by the Neuroscan system (Compumedics, Australia). Kindling stimulations were all conducted during wakefulness, similarly to our previous studies.^[^ ^12^ ^]^ The after‐discharge threshold (ADT) of each mouse was determined (monophasic square‐wave pulses, 20 Hz, 1 ms/pulse, 40 pulses) by a constant current stimulator (SEN‐7203, SS‐202J, Nihon Kohden, Japan). The stimulation current started at 40 µA and then increased by 20 µA each time, and the minimal current that produced at least 5 s after‐discharge duration (ADD) was defined as the ADT. Only mice with the ADT lower than or equal to 200 µA were used in experiments. All mice received ten suprathreshold stimulations (monophasic square‐wave pulses, 400 µA, 20 Hz, 1 ms pulse^−1^, 40 pulses, 30 min‐interval per stimulation) daily from the next day on.

Seizure severity was scored following the criteria of the Racine scale: 1. facial movement; 2. head nodding; 3. unilateral forelimb clonus; 4. bilateral forelimb clonus and rearing; and 5. rearing and falling. When mice had three successive stage 5 seizures, they were regarded as fully kindled. The EEG power recorded was calculated and analyzed offline by a software package (Labchart V8, ADInstruments, Australia).

### In Vivo Fiber Photometry Recording

A fiber photometry system (Inper, China) was used to record the fluorescence signals in freely moving mice, similarly to our previous studies.^[^ ^47^ ^]^ LED light sources 410 and 470 nm were filtered, reflected by 450 and 495 nm pass dichroic mirrors respectively, and then coupled into the optical fiber using a 20× objective lens and fiber launch. An optical fiber guided the light between the commutator and the implanted optical fiber cannula. The tip of the optical fiber transmits the light to the brain region, stimulating the astrocytes or neurons labeled with GCaMP Ca^2+^‐sensitive fluorescent protein, or other sensitive fluorescent probes. The laser intensity at the interface between the fiber tip and the animal ranged from 0.01–0.03 mW to minimize bleaching. The collected GCaMP or other probes fluorescence was converted to voltage signals by a CMOS camera (Sampling rate: 95 fps; exposure: 10 ms). Data were further analyzed by MATLAB (version R2021a, MathWorks, USA). The values of fluorescence change were shown as ΔF/F with the following equation: (ΔF/F) = (F − F_0_)/F_0_, among which F represented the current value of the signal, and F_0_ represented the average value of baseline signals. The data were presented as a time‐related peri‐event plot and heatmap. Specifically, to evaluate the Ca^2+^ activity of astrocytes during the active period, the signals were recorded during a 3 day fear conditioning test. F_0_ represented the average value of baseline signals between ‐5 and 0 s. To evaluate the Ca^2+^ activity during freezing, the signals were recorded during a 3 day fear conditioning test, F_0_ represented the average value of baseline signals between −2 and 0 s.

### Chemogenetic Manipulation

For chemogenetic manipulation, clozapine N‐oxide (CNO) (Abcam, Cat# Ab120072; dissolved in 0.9% NaCl saline), at the dose of 1 or 3 mg kg^−1^ body weight, was injected (i.p.) into mice expressing hM3Dq‐mCherry. Behavioral tests started 30 min after injection.

### Immunofluorescence

Mice were deeply anesthetized with pentobarbital sodium and perfused with 0.01 M phosphate buffer saline (PBS), followed by 4% paraformaldehyde in PBS (PFA, wt/vol). After the perfusion, the brain was removed and post‐fixed in 4% PFA in PBS at 4 °C overnight and then dehydrated in a 30% aqueous solution of sucrose (wt/vol) at 4 °C for 48 h. Brain tissue sections (30 µm) were cut in a cryostat (CM 3050S, Leica) and stored in PBS at 4 °C for further use. For immunostaining, brain section non‐specific epitopes were blocked with 5% normal donkey serum and 0.3% Triton X‐100 in PBS (vol/vol) for 1.5 h at room temperature and then incubated overnight at 4 °C with the following primary antibodies: anti‐GFAP antibody (mouse, 1:400, G3893, Sigma), anti‐NeuN (rabbit, 1:400, MABN140, Millipore), anti‐cFos antibody (rabbit, 1:2000, 226008, Synaptic Systems), anti‐CaMKIIα antibody (rabbit, 1:500, ab52476, Abcam). After washing, the sections were incubated with Alexa Fluor 488/594/647 secondary antibody (1:1000, ab150113/ ab150076/ ab150119, Abcam) for 90 min at room temperature. After being washed 3 times with PBS for 15 min, the sections were rinsed and mounted on microscope slides using an anti‐fluorescence quencher containing DAPI (Yeasen Biotech Co., Ltd., China). Finally, fluorescence images were acquired by a laser confocal microscope (TCS SP8, Leica) or an Olympus microscope (BX61).

### Statistical Analyses

Data were presented as means ± SEM. Data statistics and graph generation were performed using GraphPad Prism 9, with specific statistical analysis methods detailed in the figure legends. The normality of data in each group was tested using the Kolmogorov‐Smirnov test. For comparisons between two groups, conforming to the normality, either the Student's unpaired *t*‐test or Student's paired *t*‐test for significance analysis. Two‐way ANOVA was employed for significance analysis of mouse conditioned fear learning curves and contextual/cued memory retrieval curves. For comparisons involving more than two groups, One‐way ANOVA was used for significance analysis. Specifically, ordinary One‐way ANOVA was applied when both the Gaussian distribution and homogeneity of variance (sphericity) were met; while Repeated‐measures One‐way ANOVA was used when the Gaussian distribution was met but homogeneity of variance was not; Kruskal‐Wallis test was applied when normal distribution was not met but homogeneity of variance was; and Friedman test was adopted when neither normal distribution nor sphericity was met. When conducting significance post‐hoc tests between the groups, Tukey's multiple comparisons test or Šídák's multiple comparisons test was used. Significance was reported in the figure legends. For all analyses, *P*<0.05 was considered statistically significant.

## Conflict of Interest

The authors declare no conflict of interest.

## Author Contributions

YY.S., J.X., Y.S., and Y.L. contributed equally to this paper. Y.W., Z.C. and YY.S. designed the study and wrote the manuscript. Y.W., YL.L., YY.S., J.X., YH.S., ZS.L., F.F. and HM.C. analyzed the data. YY.S., J.X., YH.S., JL.C. and L.Y. performed the behavioral tests, mouse perfusions and molecular biological experiments. VP and YL.L. reviewed and edited the manuscript. All authors contributed to discussion and analysis of the results.

## Supporting information



Supporting Information

## Data Availability

The data that support the findings of this study are available from the corresponding author upon reasonable request.
