# Supplementary material for: Subicular Astrocytes Govern Seizure‐Impaired Fear Memory
Source: Adv Sci (Weinh). 2025 Nov 20;13(7):e10818. doi: 10.1002/advs.202510818 (PMC12866762; doi:10.1002/advs.202510818)
Supplement: Supplementary file 1 — Supporting Information [file ADVS-13-e10818-s001.docx]

Supporting Information


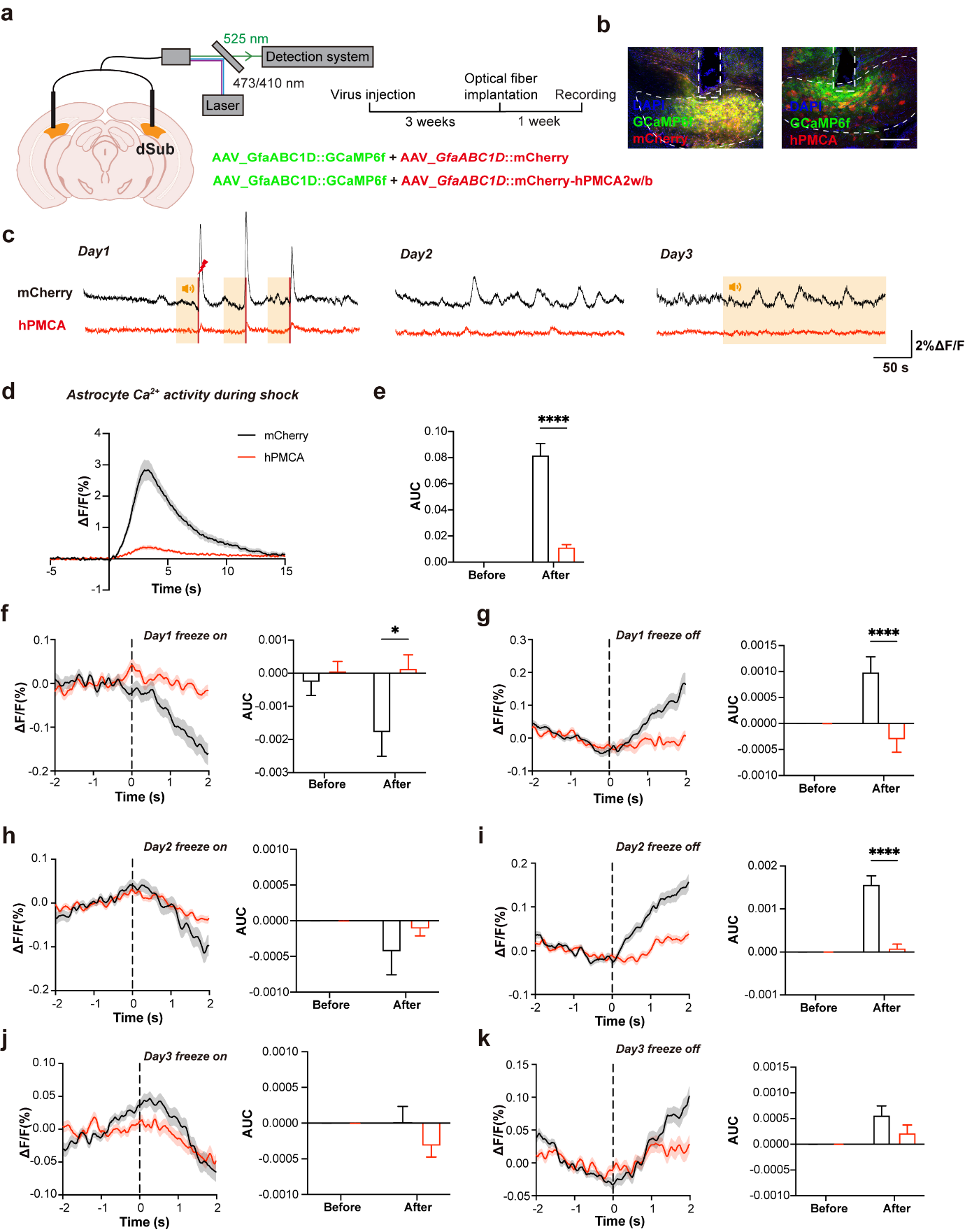


**Figure S1. hPMCA2w/b effectively reduces astrocytic Ca²⁺ signaling.** (a) Schematic of the astrocytic inhibition experiment with cytosolic Ca^2+^ recordings. Mice were injected with AAV_GfaABC1D::GCaMP6f and AAV_GfaABC1D::mCherry-hPMCA2w/b (Control: AAV_GfaABC1D::mCherry) in the dSub. Three weeks later, an optic fiber was implanted above the dSub. After the one-week recovery, mice underwent fear conditioning while recording astrocytic Ca^2+^ levels using photometry. (b) Left, representative fluorescence image of GCaMP6f (green), DAPI (blue) and mCherrry (red) in the dSub (dashed contour). Right, representative fluorescence image of GCaMP6f (green), DAPI (blue) and hPMCA-mCherrry (red) in the dSub (dashed contour). Scale bar: 200 μm. (c) Representative Ca^2+^ time series (ΔF/F percentage) for learning, contextual recall and cued recall, the orange rectangle represents the time range of sound stimulation, the vertical red lines represent the time range of foot shock. (d) Average Ca^2+^ photometry signal from astrocytes during day1 foot shocks, the timing of which is indicated by the vertical line and electrical bolt. (e) Quantification of Ca^2+^ signal as area under the curve (AUC) 5 s before the shock and 5 s after the shock (*****P*<0.05, two-way ANOVA test with Šídák's multiple comparisons test). (f, h, j) Left, Average neuronal Ca^2+^ photometry signal during the freezing initiation, indicated by the vertical dashed line. Right, Quantification of such AUC 2 s before and 2 s after the freezing initiation (**P*<0.05, two-way ANOVA test with Šídák's multiple comparisons test). (g, i, k) Left, Average neuronal Ca^2+^ photometry signal during the freezing termination. Right, Quantification of such AUC 2 s before and 2 s after freezing termination (*****P*<0.0001, two-way ANOVA test with Šídák's multiple comparisons test). N=7 for mCherry group, n=5 for hPMCA2w/b group. Data are presented as mean ± SEM.


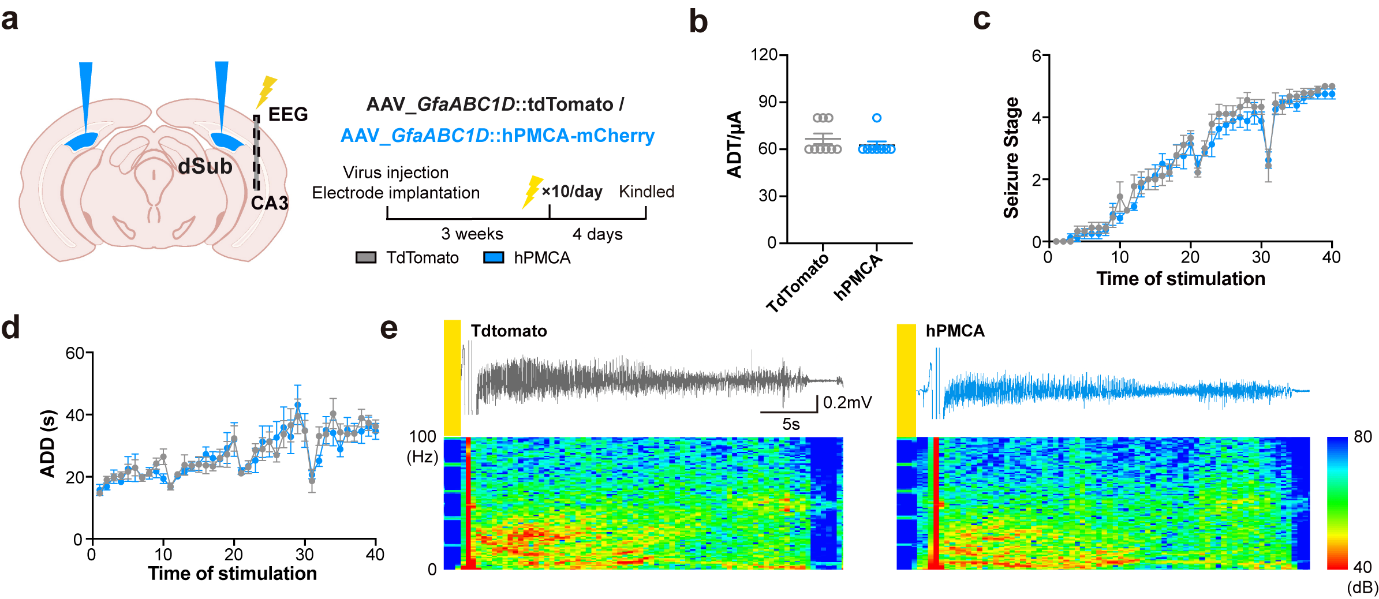


**Figure S2. Suppression of subicular astrocytes Ca^2+^ dynamics does not affect seizure susceptibility.** (a) Left, schematic of AAV5_*GfaABC1D*:: hPMCA2w/b-mCherry or AAV2/5_*GfaABC1D*::tdTomato (Control virus) bilateral injection in dorsal subicula (dSub). Dual electrode implantation in the right CA3. Right, schematic of the experiment. (b) After-discharge threshold (ADT), i.e. the minimal current intensity that could cause discharge of nerve impulses after kindling, is similar between two groups. (c, d) Lack of effects of suppression of subicular astrocytes with hPMCA-mCherry on the seizure stages and after-discharge durations (ADDs). (e) Representative CA3 EEG traces (top) and power spectrogram (bottom; time on the x-axis corresponds to that of the above trace) during generalized seizures. n=8 for tdTomato group, n=8 for hPMCA group. Data are presented as mean ± SEM.


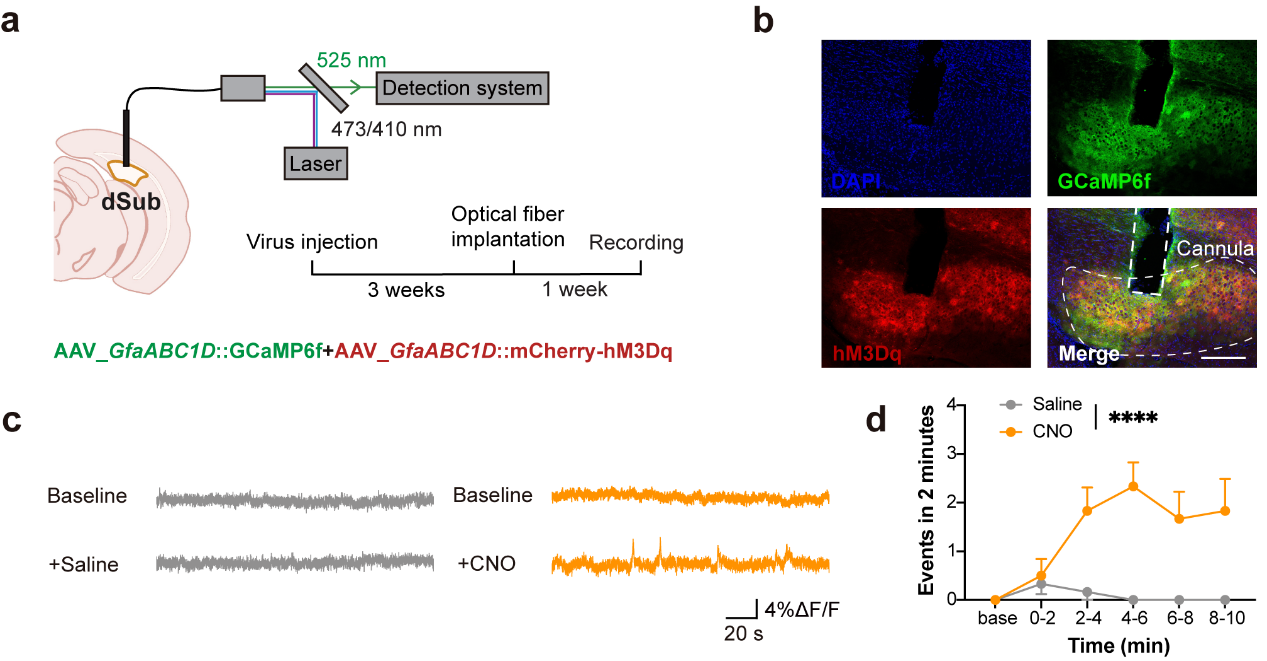


**Figure S3.** **Activation of astrocytic with Gq-DREADDs in the dSub increases astrocytic calcium levels.** (a) Schematic of the astrocytic Gq activation experiment with Ca^2+^ recordings. Mice were injected with AAV_*GfaABC1D*::hM3Dq-mCherry and AAV_*GfaABC1D*::GCaMP6f in the right dSub. Three weeks later, an optic fiber was implanted in the dSub. After the one-week recovery, performed fiber photometry. (b) Representative fluorescence image of GCaMP6f (green), DAPI (blue) and hM3Dq-mCherrry (red) in the dSub. Scale bar: 200 μm. (c) Representative astrocytic Ca^2+^ signaling between 2 groups. (d) The curve of events in 2 minutes during the bilateral Gq activation of astrocytes in dSub (*****P*<0.0001, two-way ANOVA test). N=6 for saline group, n=6 for CNO group. Data are presented as mean ± SEM.

**
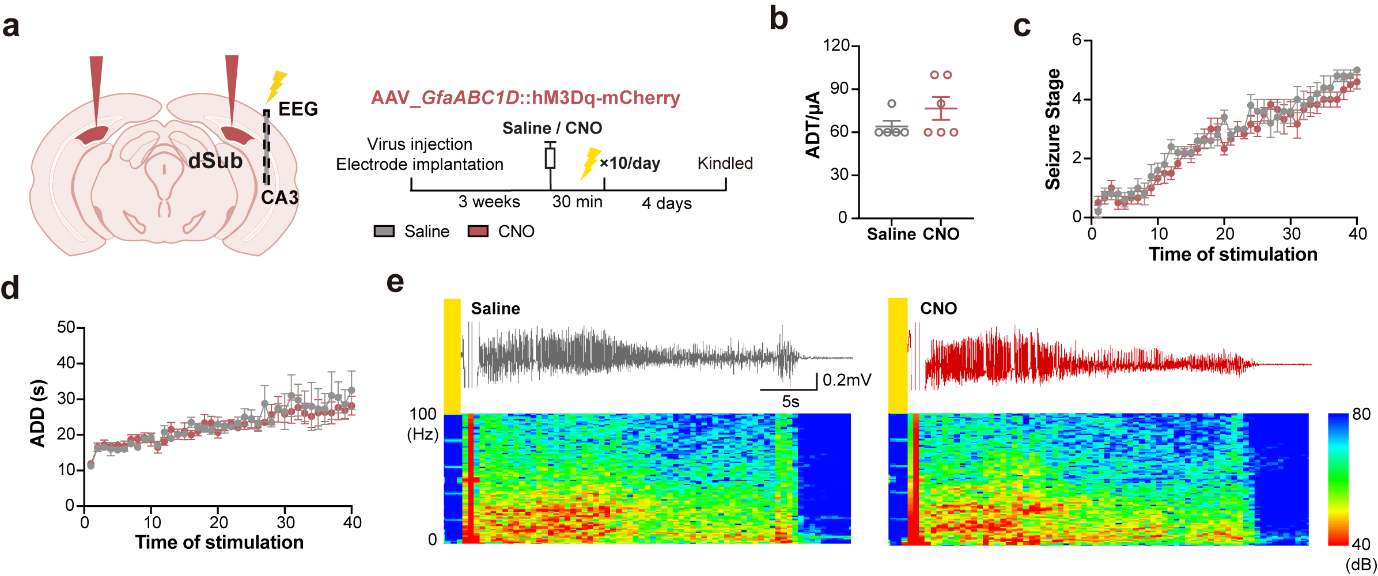
**

**Figure S4. Activation of subicular astrocytes Ca^2+^ dynamics does not affect seizure susceptibility.** (a) Left, schematic of rAAV2/5_*GfaABC1D*::hM3Dq-mCherry bilateral injection in dSub and electrode implantation in the right CA3 region. Right, schematic of the experiment. CNO, clozapine N-oxide activates hM3Dq; Saline is used as control. (b) ADT for both groups. (c, d) Lack of effects by activation of subicular astrocytes with hM3Dq + CNO (vs hM3Dq + saline as control) on the seizure stages and ADDs. (e) Representative CA3 EEGs (top) and power spectrogram (bottom) during generalized seizures, n=5 for Saline group, n=6 for CNO group. Data are presented as mean ± SEM.


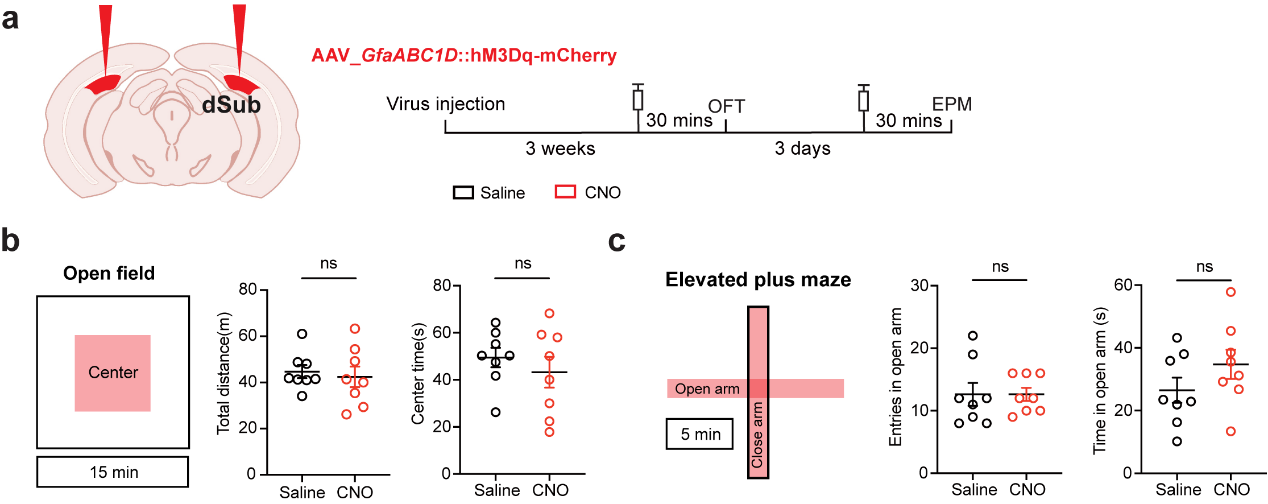


**Figure S5.** **hM3Dq activation of subicular astrocytes has no impact on locomotor or anxiety-like behavior.** (a)Left, schematic of rAAV2/5_*GfaABC1D*::hM3Dq-mCherry bilateral injection in dorsal subiculum (dSub); Right, Time line of the astrocyte Gq activation experiment in Open Field Test (OFT) and Elevated Plus Maze (EPM). Saline or CNO was administered to mice 30 minutes before behavioral tests. (b) Schematic illustration of OFT (left) with corresponding quantification of total distance traveled and time spent in the center during the 5-minute session (right). (c) Schematic illustration of EPM with corresponding quantification of open arm entries and total distance traveled in the open arms (right).

**
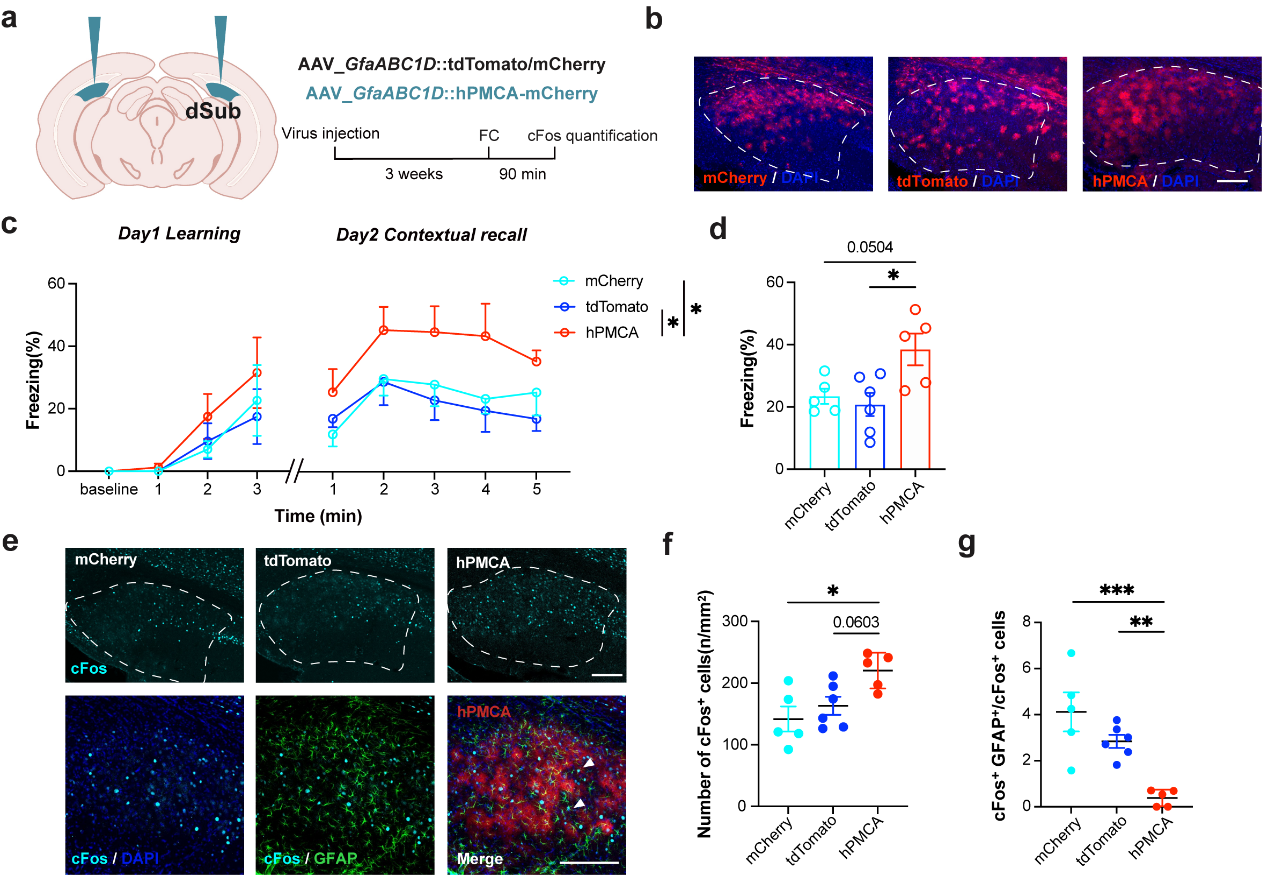
**

**Figure S6.** **hPMCA is sufficient to reduce the expression of cFos in astrocytes during memory retrieval.** (a) Left, schematic of AAV_*GfaABC1D*::hPMCA2w/b-mCherry, AAV_*GfaABC1D*::tdTomato, or AAV_*GfaABC1D*::mCherry bilateral injection in dorsal subiculum. Right, schematic of the experiment. FC, fear conditioning. (b) Representative images showing viral expression of hPMCA2w/b-mCherry, tdTomato and mCherry with cell nuclei counterstained with DAPI (blue) in dorsal subiculum (dashed contour). Scale bar: 200 μm. (c) The curve of freezing levels during the fear conditioning and contextual memory retrieval (**P*<0.05, two-way ANOVA test with Tukey's multiple comparisons test). (d) Percentage of freezing in contextual fear memory tested on the Day 2 (**P*<0.05, Brown-Forsythe ANOVA test with Dunnett's T3 multiple comparisons test). (e) Representative fluorescence images of immunofluorescence co-staining of cFos (teal) and GFAP (green). Cell nuclei are counterstained with DAPI (blue). Scale bar: 200 μm. Arrowheads indicate the individual cFos co-label with GFAP. (f) Quantification of the cFos^+^ cells between 3 groups (**P*<0.05, Brown-Forsythe ANOVA test with Dunnett's T3 multiple comparisons test). (g) Quantification of the proportion of cFos^+^ cells in GFAP^+^ cells (***P*<0.01, ****P*<0.001, Brown-Forsythe ANOVA test with Dunnett's T3 multiple comparisons test). N=5 for hPMCA2w/b group, n=6 for tdTomato group, n=5 for mCherry group. Data are presented as mean ± SEM.


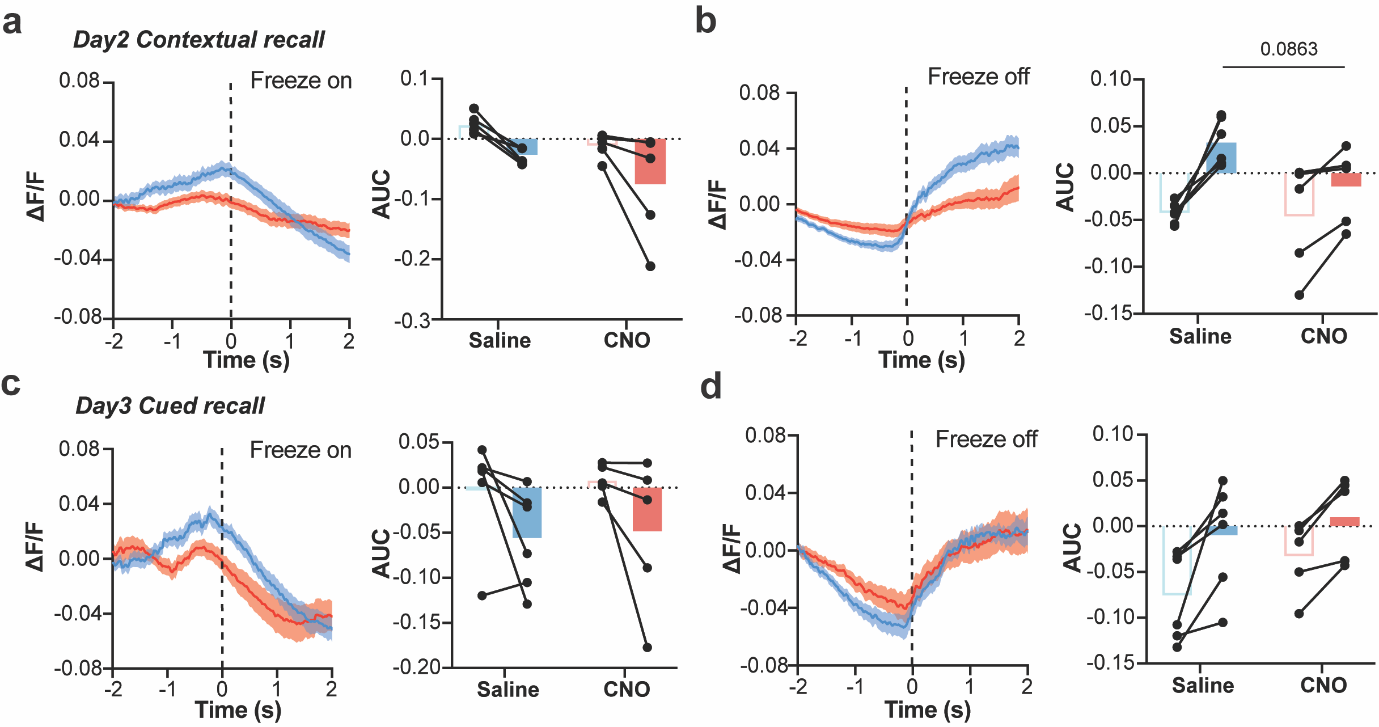


**Figure S7. Activation of Gq signaling pathway in subicular astrocytes lacks any significant effect on Ca^2+^ dynamics in local CaMKIIα^+^ neurons during contextual and cued recalls.** Experiment conditions as in Fig. 5e-i. (a) Left, Average neuronal Ca^2+^ photometry signal during the day 2 freezing initiation. Right, Quantification of area under the curve (AUC) 2 s before and 2 s after freezing initiation. (b) Right, Average neuronal Ca^2+^ photometry signal during the day 2 freezing termination. Right, Quantification of AUC 2 s before and 2 s after freezing initiation. (c-d) Left, Average neuronal Ca^2+^ photometry signal during the day 3 freezing initiation (c) and termination (d). Right, Quantification of AUC 2 s before and 2 s after freezing initiation (c) and termination (d). N=6 for Saline group, n=5 for CNO group. Data are presented as mean ± SEM.


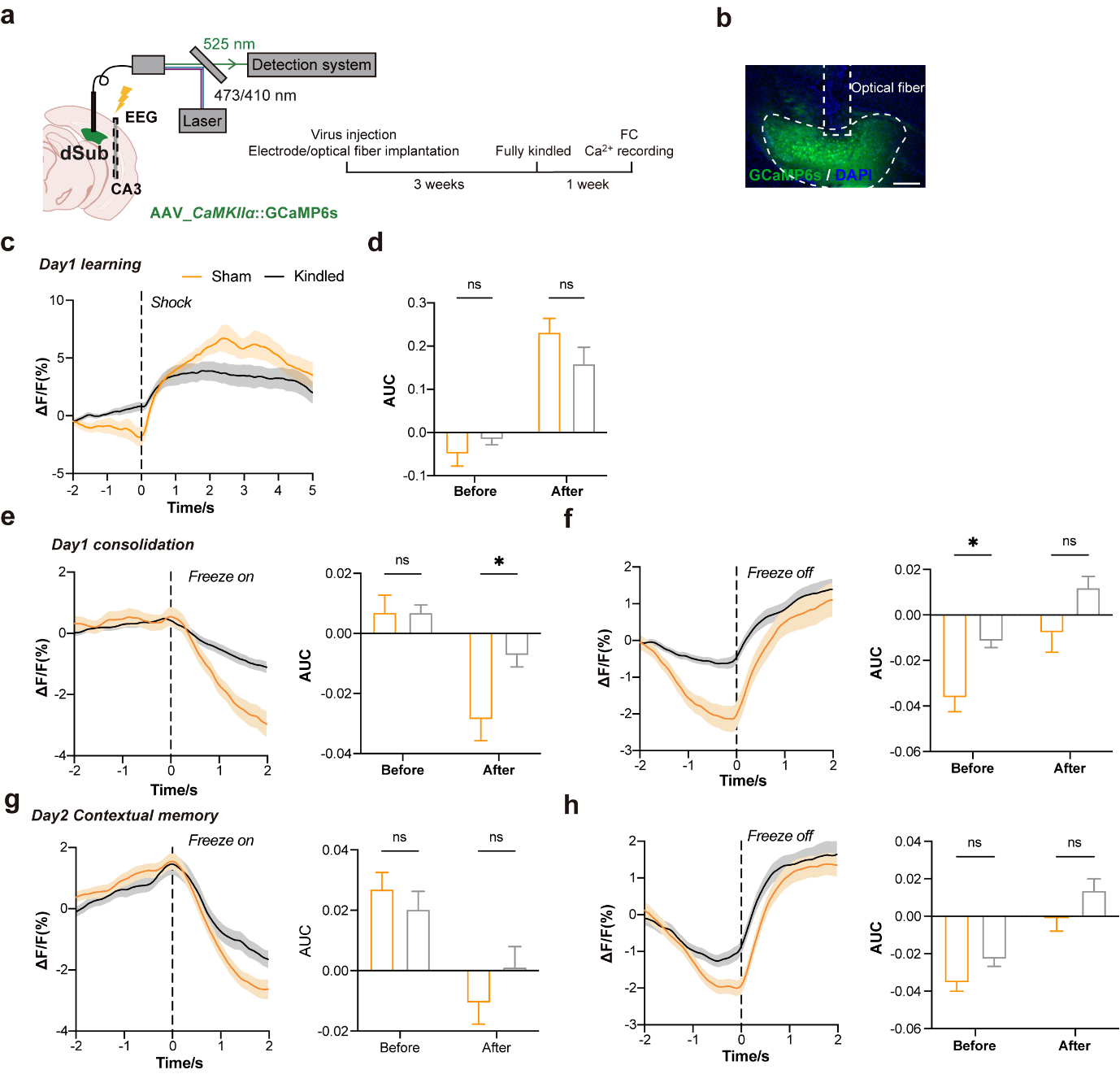


**Figure S8. Impaired CaMKIIα^+^ neuronal activity in kindled epileptic mice.** (a) Left, schematic of AAV-*CaMKIIα*::GCaMP6s viral injection into dSub and optic fiber implantation above the dSub, and electrode, dual for stimulation and EEG recordings, implantation in the right ventral CA3 region; Right, schematic of the experiment. (b) Representative image showing optic fiber (vertical dashed rectangle) placement and GCaMP6s expression (green) in the dSub (dashed contour). Scale bar: 200 μm. (c) Average Ca^2+^ photometry signal from neurons during day1 foot shocks, the timing of which is indicated by the vertical line and electrical bolt. (d) Quantification of neuronal Ca^2+^ signal as area under the curve (AUC) 5 s before the shock and 5 s after the shock. (e, g) Left, Average neuronal Ca^2+^ photometry signal during the Day1 and Day2 freezing initiation, indicated by the vertical dashed line. Right, Quantification of such AUC 2 s before and 2 s after the freezing initiation (*P<0.05, two-way ANOVA test with Šídák's multiple comparisons test). (f, h) Left, Average neuronal Ca^2+^ photometry signal during the Day 1 and Day2 freezing termination. Right, Quantification of such AUC 2 s before and 2 s after freezing termination (*P<0.05, two-way ANOVA test with Šídák's multiple comparisons test), n=4 for Sham group, n=3 for Kindled group. Data are presented as mean ± SEM.

**
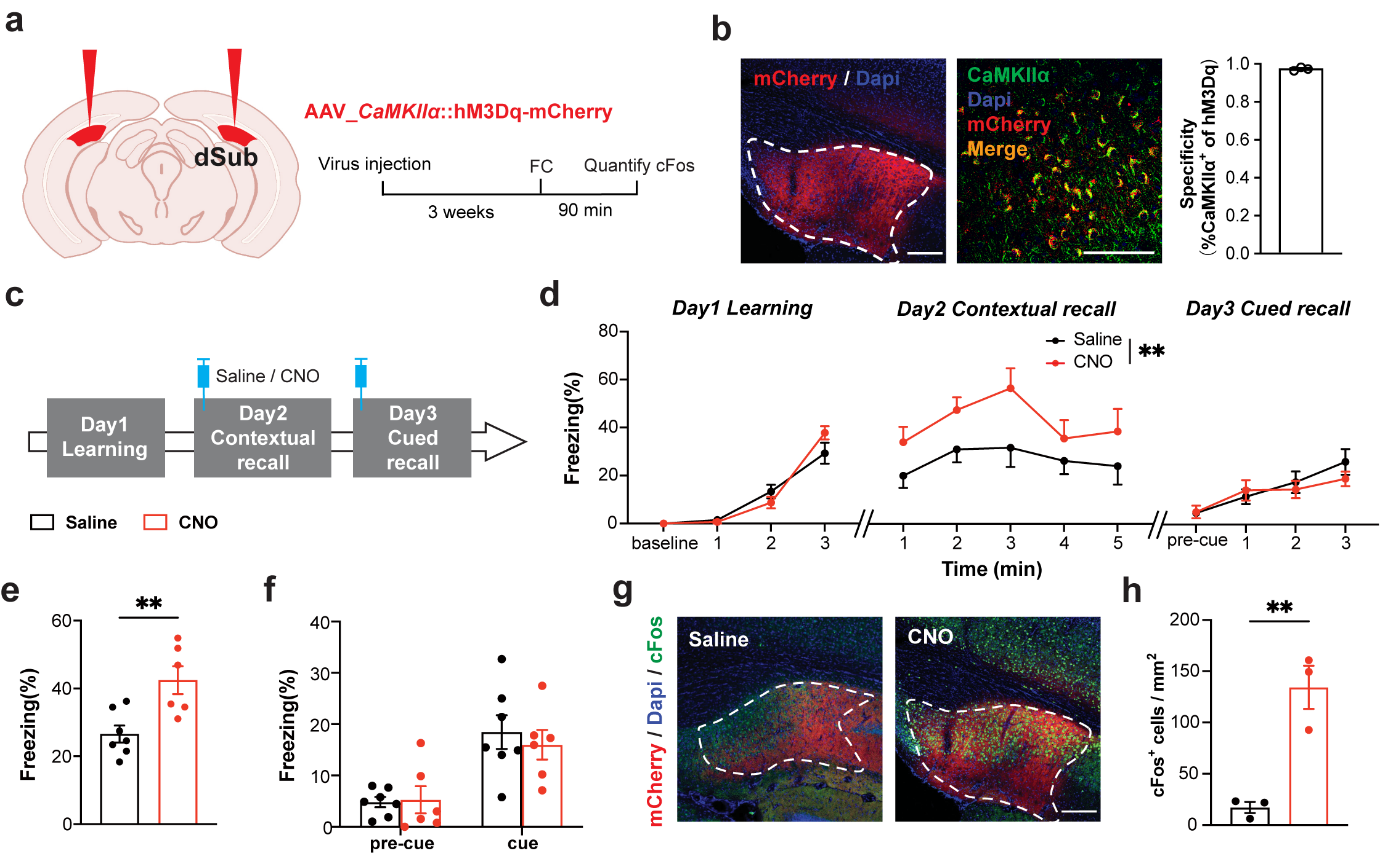
**

**Figure S9. Activation of subicular CaMKIIα^+^ neurons enhance the contextual memory retrieval.** (a) Left, schematic of rAAV2/R_*CaMKIIα*::hM3Dq-mCherry bilateral injection in the dorsal subicula (dSub) ; Right, timeline of the experiment. FC, fear conditioning. (b) Images: Cell nuclei are stained by DAPI (blue). Left, a typical image for dSub (dashed contour) showing expression of hM3Dq-mCherry. Scale bar: 200 μm; Right, a fluorescence image of hM3Dq-mCherry (red) and immunofluorescence co-staining of CaMKIIα (green). Scale bar: 100 μm. Graph: Co-localization analysis of CaMKIIα^+^ neurons and hM3Dq-mCherry shows neuron-specific expression of hM3Dq as this signal co-localizes almost to its entirety with that of neurons. (n=3). (c) Schematic of the 3-day fear conditioning test with CaMKIIα::hM3Dq CNO-mediate activation during fear memory retrievals. Saline acts as control. (d) The curve of freezing levels during the 3-day fear conditioning with the bilateral Gq activation of neurons in dSub, as outlined in (c) (***P*<0.01, two-way ANOVA test), n=7 for Saline group (black traces and open bars in e), n=6 for CNO group (red traces and red open bars in e). (e) The percentage of freezing in contextual fear memory tested on the day 2 in (d) (**P<0.01, unpaired t-test). (f) The percentage of freezing in cued fear memory recall tested on the day. 3 (g) Representative image of cFos expression in the dSub at day 2 after testing contextual recall. Cell nuclei are stained by DAPI (blue). Scale bar: 200 μm. (h) Quantification of the number of cFos in the subiculum at day 2 after testing contextual recall (**P<0.01, unpaired t-test), n=3 for Saline group (open black bars), n=3 for CNO group (open red bars), 3 slices for each mouse. Data are presented as mean ± SEM.

**
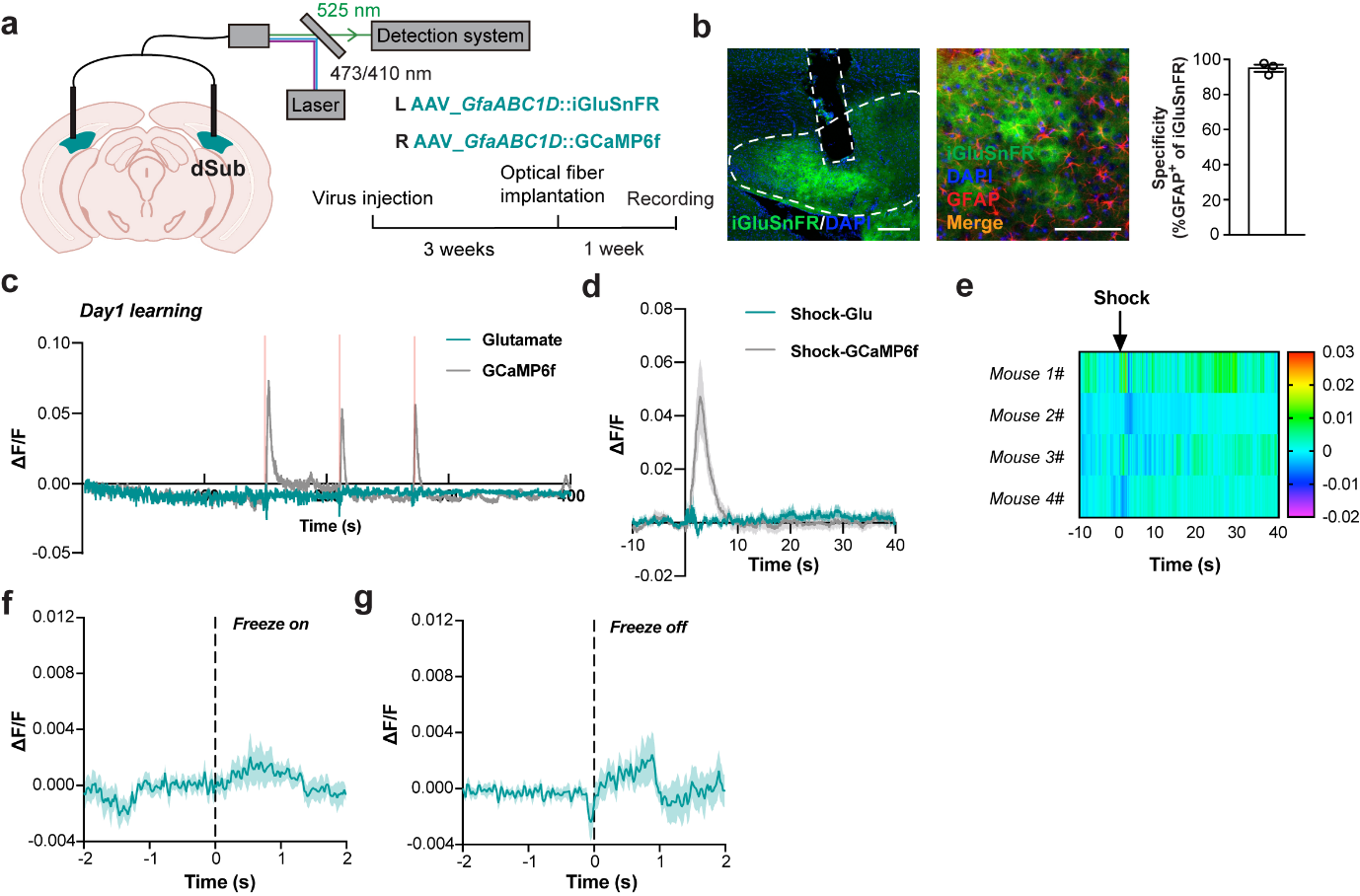
**

**Figure S10. Subicular astrocytic Ca^2+^ dynamics during fear acquisition do not result in associated extracellular glutamate dynamics.** (a) Left, schematic of rAAV2/9_*GfaABC1D*::iGluSnFR(A184S) injection in the left (L) dorsal subiculum (dSub), and AAV5_*GfaABC1D*::GCaMP6f injection in the contralateral, right (R) dSub; Right, schematic of the experiment. (b) Images: Cell nuclei are stained by DAPI (blue). Left, a typical image a dSub (dashed contour), the astrocyte of which express iGluSnFR (Green). The optic fiber location is indicated by the dashed vertical rectangle. Scale bar: 200 μm; Right, a fluorescence image of iGluSnFR (green) and immunofluorescence co-staining of GFAP (red). Scale bar: 100 μm. Graph: Co-localization analysis of GFAP^+^ astrocytes and iGluSnFR expression shows astrocyte-specific expression of iGluSnFR as this signal co-localizes almost to its entirety with that of astrocytes; n=3. (c) Representative time-lapse of astrocytic Ca^2+^ (GCaAMP6f) and extracellular glutamate (iGluSnFR) signal (ΔF/F percentage) during conditioned fear learning; 0.5 mA foot shocks occurred at the 148, 208 and 268 s time points, as indicated by vertical orange lines. (d) Traces of astrocytic Ca^2+^ and extracellular glutamate dynamics after foot shock (t=0), n=4, 3 trials per mouse. (e) Heatmap of extracellular glutamate levels. Foot shock is indicated by the arrow. Right, vertical bar displays a pseudo color representation of ΔF/F ranging from -0.02 to 0.03; n=4, 3 trials per mouse. (f, g) Quantification of extracellular glutamate dynamics at the initiation or termination of freezing behavior on the learning day 1, with each event occurring at the dashed line (time=0). 10 trials from 4 mice. Data are presented as mean ± SEM.


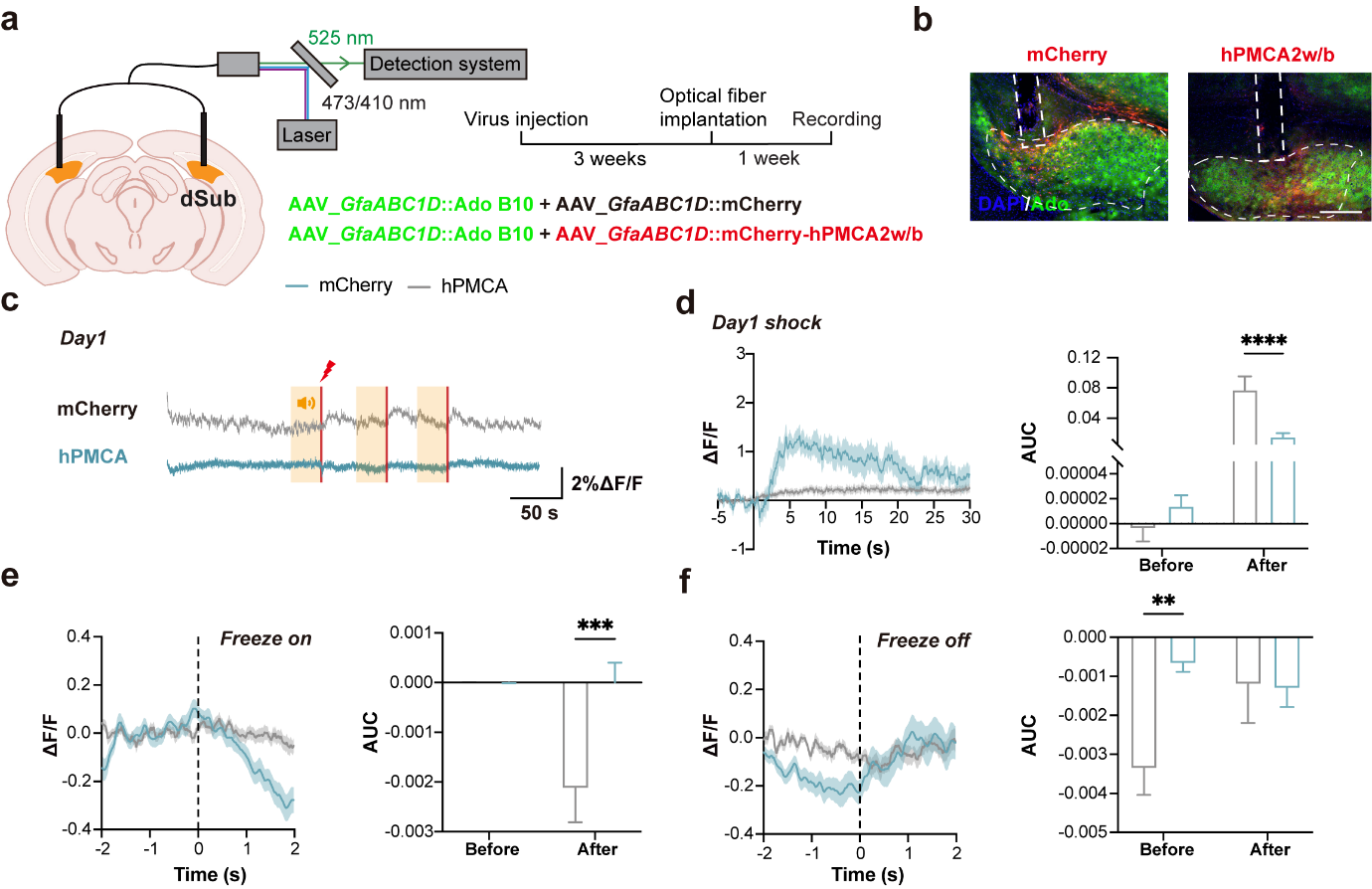


**Figure S11. hPMCA2w/b effectively reduces astrocytic adenosine signaling.** (a)Schematic of the astrocytic inhibition experiment with adenosine signaling recordings. Mice were injected with AAV_*GfaABC1D*::Ado_B10 and AAV_GfaABC1D::mCherry-hPMCA2w/b (Control: AAV_GfaABC1D::mCherry) in the dSub. Three weeks later, an optic fiber was implanted in the dSub. After the one-week recovery, mice underwent fear conditioning while recording adenosine signaling using photometry. (b) Left, representative fluorescence image of Ado (green), DAPI (blue) and mCherrry (red) in the dSub (dashed contour). Right, representative fluorescence image of Ado (green), DAPI (blue) and hPMCA-mCherry (red) in the dSub (dashed contour). Scale bar: 200 μm. (c) Representative Ado time series (ΔF/F percentage) for the learning day, the orange rectangle represents the time range of sound stimulation, the vertical red lines represent the time range of foot shock. (d) Left, average Ado photometry signal from astrocytes during day1 foot shocks. Right, quantification of Ado signal as area under the curve (AUC) 5 s before the shock and 5 s after the shock (*****P*<0.05, two-way ANOVA test with Šídák's multiple comparisons test). (e) Left, Average neuronal Ado photometry signal during the day1 freezing initiation, indicated by the vertical dashed line. Right, Quantification of such AUC 2 s before and 2 s after the freezing initiation (****P*<0.001, two-way ANOVA test with Šídák's multiple comparisons test). (f) Left, Average neuronal Ado photometry signal during the day1 freezing termination. Right, Quantification of such AUC 2 s before and 2 s after freezing termination (***P*<0.01, two-way ANOVA test with Šídák's multiple comparisons test, n=4 for mCherry group, n=6 for hPMCA2w/b group. Data are presented as mean ± SEM.


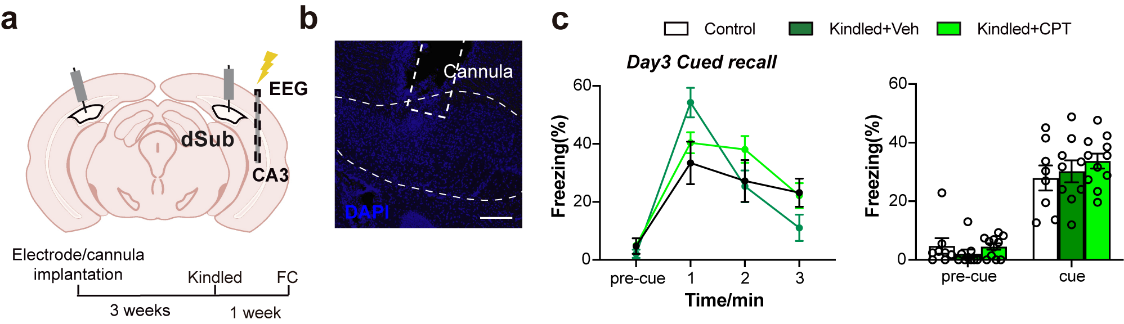


**Figure S12. Adenosine signaling pathway do not contribute to cued memory in hippocampus-kindled mice.** (a) Top, schematics of bilateral cannula implantation above dSub and electrode implantation in the right CA3; Bottom, schematic of the experiment. FC, fear conditioning. (b) A representative image of a dorsal subiculum (dashed contour) with DAPI-stained cell nuclei (blue). The cannula placement is indicated by a dashed rectangle. Scale bar: 200 μm. (c) Percentage of freezing in cued fear memory tested on the day 3, n=8 for Control (unkindled and no Veh/CPT) group, n=10 for Kindled+Veh group, n=10 for Kindled+CPT group. Data are presented as mean ± SEM.
